# Structure of the polyisoprenyl-phosphate glycosyltransferase GtrB and insights into the mechanism of catalysis

**DOI:** 10.1038/ncomms10175

**Published:** 2016-01-05

**Authors:** Chiara Ardiccioni, Oliver B. Clarke, David Tomasek, Habon A. Issa, Desiree C. von Alpen, Heather L. Pond, Surajit Banerjee, Kanagalaghatta R. Rajashankar, Qun Liu, Ziqiang Guan, Chijun Li, Brian Kloss, Renato Bruni, Edda Kloppmann, Burkhard Rost, M. Chiara Manzini, Lawrence Shapiro, Filippo Mancia

**Affiliations:** 1 Department of Physiology and Cellular Biophysics, Columbia University, New York, New York 10032, USA; 2 Department of Biochemistry and Molecular Biophysics, Columbia University, New York, New York 10032, USA; 3 Department of Pharmacology and Physiology, George Washington University, Washington, District of Columbia 20037, USA; 4 Department of Integrative Systems Biology, George Washington University, Washington, District of Columbia 20037, USA; 5 NE-CAT and Department of Chemistry and Chemical Biology, Cornell University, Argonne National Laboratory, Argonne, Illinois 60439, USA; 6 New York Structural Biology Center, X4 Beamlines, Brookhaven National Laboratory, Upton, New York 11973, USA; 7 Department of Biochemistry, Duke University Medical Center, Durham, North Carolina 27710, USA; 8 New York Consortium on Membrane Protein Structure, New York Structural Biology Center, New York, New York 10027, USA; 9 Department of Informatics, Bioinformatics and Computational Biology, Garching 85748, Germany; 10 Institute for Advanced Study (TUM-IAS), TUM (Technische Universität München), Garching 85748, Germany

## Abstract

The attachment of a sugar to a hydrophobic polyisoprenyl carrier is the first step for all extracellular glycosylation processes. The enzymes that perform these reactions, polyisoprenyl-glycosyltransferases (PI-GTs) include dolichol phosphate mannose synthase (DPMS), which generates the mannose donor for glycosylation in the endoplasmic reticulum. Here we report the 3.0Å resolution crystal structure of GtrB, a glucose-specific PI-GT from *Synechocystis*, showing a tetramer in which each protomer contributes two helices to a membrane-spanning bundle. The active site is 15 Å from the membrane, raising the question of how water-soluble and membrane-embedded substrates are brought into apposition for catalysis. A conserved juxtamembrane domain harbours disease mutations, which compromised activity in GtrB *in vitro* and in human *DPM1* tested in zebrafish. We hypothesize a role of this domain in shielding the polyisoprenyl-phosphate for transport to the active site. Our results reveal the basis of PI-GT function, and provide a potential molecular explanation for *DPM1*-related disease.

## Abstract (web summary)

Polyisoprenyl-glycosyltransferases (PI-GTs) catalyse the addition of sugar to lipid carriers, which is the first step in the production of sugar donors for glycosylation. Here Ardiccioni *et al.* present the structure of a bacterial PI-GT and propose a mechanistic basis for sugar transfer.

## Graphical abstract

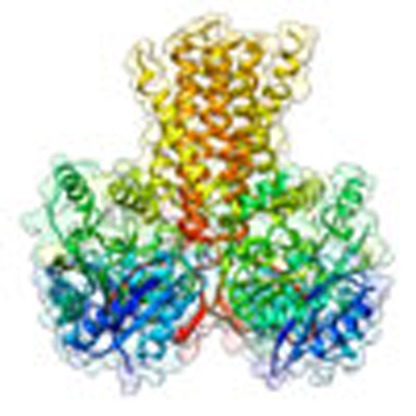

In humans and bacteria alike, the first step in all glycosylation reactions that take place in outer cellular compartments involves the attachment of a sugar in the cytoplasm to a hydrophobic carrier molecule to generate a sugar donor molecule for transmembrane (TM) export^1^. In eukaryotes, such donors are necessary for processes including N- and O-linked protein glycosylation and glycophosphatidylinositol anchoring. In Gram-negative bacteria, they contribute to the biosynthesis of the cell wall, and its modifications that underlie serotype conversion, a bacterial mechanism for evasion of the host immune response^2^. Sugar attachment to hydrophobic carrier molecules for transport across membranes is catalysed by polyisoprenyl-glycosyltransferases (PI-GTs), integral membrane enzymes conserved across all kingdoms of life^3^. Bacterial PI-GTs utilize undecaprenyl-phosphate (UndP) as the sugar carrier, whereas their eukaryotic and archaeal counterparts use the related dolichol phosphate (DolP)^3^. Mutations in the human PI-GT dolichol phosphate mannose synthase (DPMS) catalytic subunit *DPM1* (refs 4, 5)—the enzyme that charges DolP with mannose for protein glycosylation—cause congenital disorders of glycosylation^1^,^6^,^7^,^8^ type 1e resulting in severe phenotypes including developmental delay and vascular abnormalities^9^. PI-GTs have a predicted two-domain architecture composed of a cytosolic amino-terminal catalytic domain and a TM region, but their functional assemblies, detailed structures and catalytic mechanisms remain unknown.

Here we report the first structure and functional characterization of a representative PI-GT, GtrB from *Synechocystis* sp. PCC6803 (ref. 10; GtrB_*Syn*_) to 3.0 Å resolution. We describe enzyme assays that show that recombinant GtrB_*Syn*_ expressed in *Escherichia coli* attaches a glucose molecule to UndP, a key reaction for immune evasion by aggressive pathogens such as *Shigella flexneri* and *Salmonella enterica*^11^,^12^. GtrB is a tetramer in which each protomer contributes two TM helices to a membrane-spanning bundle. The soluble glycosyltransferase (GT) domains are separated from the TM helices by a juxtamembrane region that positions the active site 15 Å below the membrane surface. This structure, together with results of functional mutagenesis on GtrB_*Syn*_ and human DPM1 in a zebrafish model, provides the framework to understand how PI-GTs work in both prokaryotes and eukaryotes.

## Results

### Crystallization and structure determination

To determine the structure of a representative member of the PI-GT family, we followed a structural genomics approach, and screened 45 prokaryotic PI-GTs, identified via a bioinformatics analysis, for expression in *E. coli* and stability in detergents amenable to crystallization^13^. GtrB_*Syn*_, the PI-GT from *Synechocystis* sp. PCC6803, was the most promising. Indeed, we produced crystals of this protein, in the detergent *n*-decyl-β-D-maltopyranoside (DM), which diffracted X-rays anisotropically to 3.2/3.6 Å resolution. We determined this structure by multi-crystal single-wavelength anomalous diffraction (SAD)^14^ analysis of the selenomethionine-substituted protein. Subsequently, although probing the function of the enzyme with structure-based mutagenesis, we produced crystals of the point mutant F215A, which fortuitously diffracted isotropically to 3.0 Å, allowing us to improve the quality of the model (Table 1). F215A, which resides in a functionally relevant region of the protein, has compromised catalytic activity (Supplementary Table 1), but the structures are essentially identical, and unless indicated otherwise, we show here the better F215A model.

### Functional characterization of GtrB from *Synechocystis*

**Figure 1:**
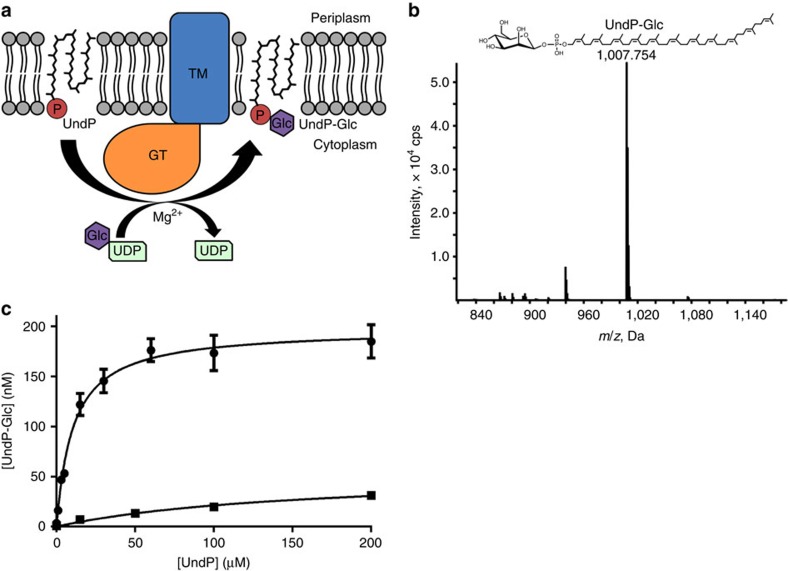
GtrB catalyses the formation of UndP-glucose *in vivo* and *in vitro*. (**a**) GtrB (TM domain in blue, GT domain in orange) catalyses the transfer of a glucose from UDP-glucose to UndP, producing UndP-glucose. (**b**) Cells overexpressing GtrB accumulate a species detected by negative ion ESI/MS at *m*/*z* of 1,007.75, corresponding to the [M-H]-ion of UndP-glucose. Its identity was further confirmed by tandem mass spectrometry (MS/MS). (**c**) When incubated with varying concentrations of UndP for 1 h, membranes prepared from GtrB-overexpressing cells accumulate UndP-glucose (UndP-Glc) in a concentration-dependent manner (circles), whereas those prepared from control cells overexpressing a membrane protein of unrelated function do not (squares). Error bars are provided as ±s.e.m., *n*=3.

GtrB was initially identified in bacteriophage-infected *Shigella flexneri* strains in a cluster of genes that mediate serotype conversion by glucosylating- and acetylating-specific residues in the O-antigen of the cell wall^11^,^15^. GtrB is a *bona fide* PI-GT as it carries out the first step in the glucosylation process, generating UndP-glucose on the cytoplasmic face of the inner membrane (Fig. 1a), which is later utilized as a glucose donor during glucosylation of the O-antigen. To assess the PI-GT function of the enzyme from *Synechocystis*, we performed mass spectrometry on lipid extracts from *E. coli* cells overexpressing GtrB_*Syn*_, revealing specific accumulation of UndP-glucose (Fig. 1b). *In vitro* assays on GtrB_*Syn*_, monitoring the UndP-specific accumulation of ^14^C in a water-insoluble phase from UDP-^14^C-glucose also confirmed the synthesis of UndP-glucose from UDP-glucose and UndP (Fig. 1c).

### The structural architecture of GtrB

**Figure 2:**
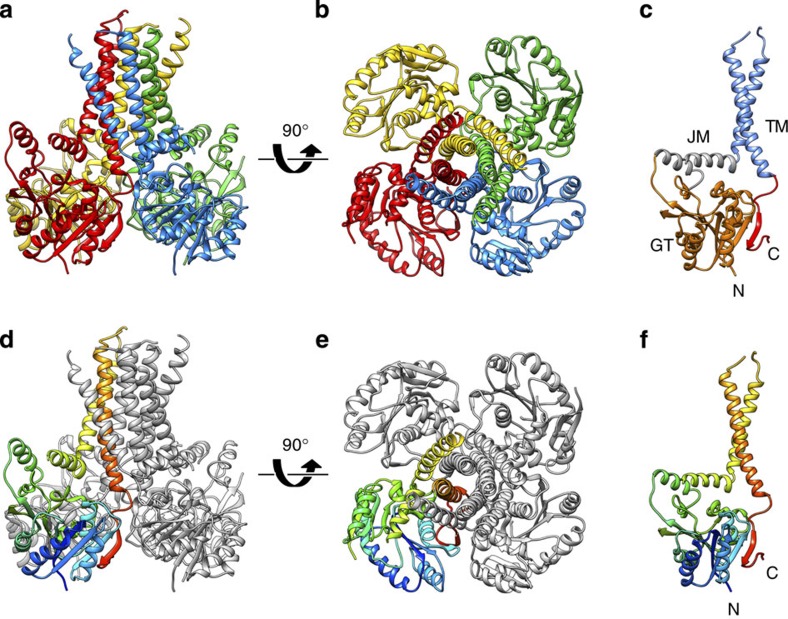
Architecture of GtrB, an integral membrane PI-GT. The tetrameric assembly of GtrB, in which each of the four polypeptide chains is represented as a ribbon drawn in a different colour, is shown here (**a**) from a position orthogonal to the TM helices and (**b**) looking down the fourfold symmetry axis of the tetramer from the extracellular side. (**c**) A single protomer is shown. The protomer is comprised of a GT-A fold GT domain (orange), two amphipathic juxtamembrane helices (grey; juxtamembrane (JM)), two TM helices (blue) and a C-terminal β-hairpin (red). (**d**,**e**) The GtrB tetramer is shown in the same two views and orientations as in **a**,**b**, with three subunits represented in grey ribbons and the fourth in rainbow colouring from the N-terminus (blue) to the C-terminus (red). (**f**) The GtrB monomer shown in the same orientation as in **c**, represented in rainbow colouring from the N-terminus (N; blue) to the C-terminus (C; red).

The structure shows that GtrB_*Syn*_ is a tetramer (Fig. 2a,b), with each protomer consisting of an N-terminal cytosolic GT domain of the GT-A fold^16^, a juxtamembrane region, two TM helices (TM1 and TM2), which form a compact stalk anchoring the intracellular domains to the membrane, and a short C-terminal beta-hairpin (Fig. 2c–f and Supplementary Fig. 1). The short beta-hairpin at the C-terminus bridges the interaction between adjacent subunits in the tetramer (Supplementary Fig. 2). Two amphipathic juxtamembrane helices (JM1 and JM2; Fig. 2c–f and Supplementary Fig. 1) lie at the cytoplasmic surface of the membrane, oriented parallel to one another, and sharing a hydrophobic interface (Supplementary Fig. 3). JM1 is located in an internal loop in the GT domain, whereas JM2 is an N-terminal extension of TM1 (Supplementary Fig. 1) analogous to the ‘slide helix’ found in potassium channel structures^17^. JM1 was first identified by analysis of the predicted secondary structure^18^, which suggested the presence of a conserved amphipathic helix located in the β5-6 loop and spanning residues 130–146. Although the density in this region is poorer than in other regions of the map (Supplementary Figs 4 and 5a), the presence of two methionine residues helped confirm the register of the sequence using selenomethionine-labelled protein crystals (Supplementary Fig. 5b).

Despite unambiguous experimental density for the majority of the molecule, electron density for the periplasmic loop that connects the TM helices is poor, leading to two possible topologies for subunit association in the tetramer (Supplementary Fig. 6). Based on our interpretation of the maps, we believe the most probable topology results in the C-terminal short beta-hairpin forming close interactions with the originating protomer (Supplementary Fig. 6a), rather than with the adjacent one (Supplementary Fig. 6b). The coordinates we have deposited leaves this ambiguity unresolved, with the protomer divided into two chains. However, for the sake of clarity, all figures showing the GtrB_*Syn*_ structure depict the topology we judge to be most probable.

### Donor and acceptor sites in GtrB

**Figure 3:**
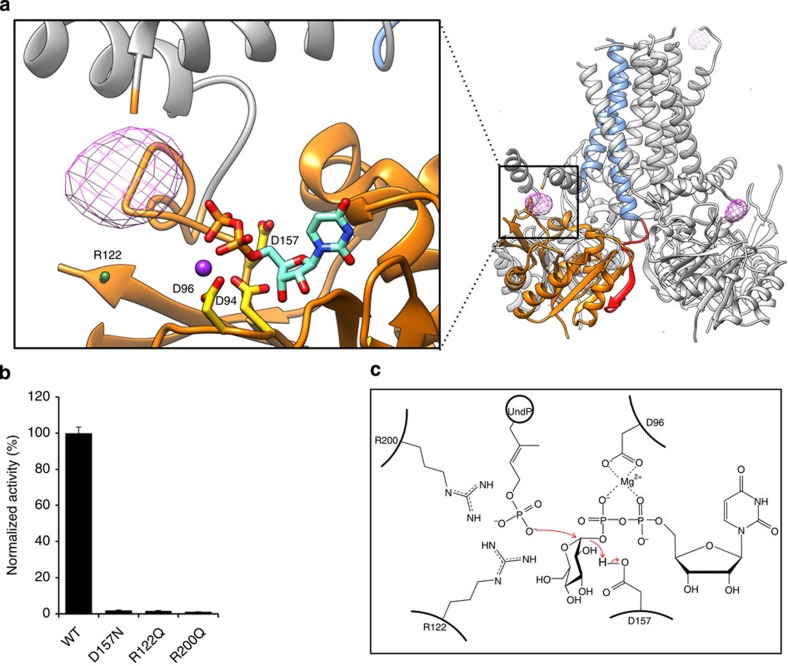
The catalytic mechanism of GtrB. (**a**) The disposition of key residues in the active site and interactions with the acceptor and donor substrates. An anomalous difference Fourier map (contoured at 5 σ above the mean) calculated from a tungstate-soaked wild-type (WT) crystal is represented as purple mesh. Residues D94, D96, R122, D157, R200 and the UDP are shown in stick representation. Mn^2+^ is represented as a purple sphere. (**b**) Mutation of key residues in the acceptor and donor sites abolishes GtrB activity. (**c**) The catalytic mechanism of GtrB does not require a catalytic base. Error bars are provided as s.e.m., *n*=3.

GtrB_*Syn*_ crystals were typically soaked in UDP-glucose before cryoprotection and freezing, as this step led to better resolution and reproducibility in the quality of the diffraction pattern. This soaking step had the additional advantage of allowing us to specifically identify the monosaccharide donor site. The UDP-glucose donor substrate binds in a shallow cleft on the surface of the cytosolic GT domain, in a similar position to that determined in other structures of GT-A fold GTs^19^ (Fig. 3a). A divalent cation mediates the interaction between the diphosphate of the UDP and a signature DXD motif^20^ (^94^Dx^96^D in GtrB_*Syn*_). The anomalous signal of manganese soaked into the crystals was used to positively identify the ion-binding site although magnesium appears to be preferred for optimal activity of the enzyme (Supplementary Fig. 7a). Despite the fact that UDP-glucose was soaked into the crystals immediately before cryoprotection and freezing, interpretable density was only observed for the UDP (Supplementary Fig. 7b), indicating that the glucose is either poorly ordered or was hydrolysed before data collection, as has previously been observed for other GTs^21^.

To identify the UndP acceptor site, we performed anomalous diffraction experiments with a GtrB_*Syn*_ crystal soaked with tungstate, a phosphate analogue with a larger X-ray anomalous scattering signal (Fig. 3a and Supplementary Fig. 8). Remarkably, the anomalous difference density overlays perfectly with the position expected from structural alignments with other GT-acceptor complexes from soluble enzymes of the GT-A fold (Supplementary Fig. 9a).

In the 3.0 Å resolution structure without tungstate, we cannot see side chain density for R122, and the segment between R197 and N208, and the limited resolution (4.5 Å) of the data with tungstate does not allow us to reliably assign previously disordered sequence in the map.

However, we can hypothesize that R122 and R200, which are universally conserved among prokaryotic and eukaryotic GTs that process lipid-phosphate acceptors (Supplementary Fig. 9b), will coordinate the acceptor. Mutation of either of these two arginines to glutamine or alanine severely compromised GtrB_*Syn*_ activity, suggesting that they play an important role in binding the phosphate of the acceptor (Fig. 3b).

### The chemistry of catalysis

The transfer of a sugar group to the acceptor molecule can occur with either inversion or retention of the stereochemistry at the anomeric carbon. The corresponding two classes of GTs are known as inverting and retaining, respectively^22^. Overall, the data suggest that GtrB is an inverting GT, based both on previous sequence-based classification and the shallow, solvent-exposed nature of the active site seen in the GtrB_*Syn*_ structure, a feature typical of inverting GTs^23^. The mechanism of catalysis, while broadly similar to that described for other inverting GTs, differs in detail due to the nature of the acceptor^24^. Unlike most GTs, which attach a saccharide donor at a neutral, protonated hydroxyl on the acceptor substrate, GtrB catalyses the attachment of glucose to the phosphate oxygen, which is expected to be deprotonated under physiological conditions. A key step in the reaction mechanism of conventional inverting GTs involves the participation of a catalytic base residue, which abstracts a proton from the acceptor, enabling nucleophilic attack by the resultant lone pair of electrons at the anomeric carbon^24^,^25^. When the acceptor is already deprotonated, it is unclear whether this step is necessary, and it seems likely that the role of a catalytic acid residue in assisting hydrolysis of the UDP-sugar donor will be more prominent. Based on structural alignment with other GT-A fold GTs and mutagenesis data showing that GtrB_*Syn*_ activity is abolished in the D157N mutant (Fig. 3b), we identified D157 as a putative catalytic acid, and propose a tentative scheme for how the reaction catalysed by GtrB may take place, and describe it here in the context of the GtrB_*Syn*_ structure (Fig. 3c).

### Bringing substrates into apposition for catalysis to occur

**Figure 4:**
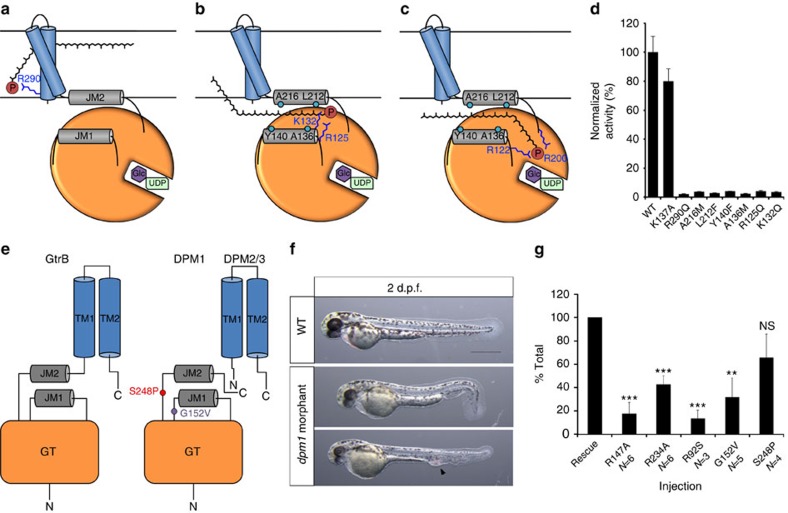
A putative mechanism for substrate translocation in GtrB and DPM1 function in a zebrafish model. (**a**) A speculative hypothesis is that the phosphate headgroup of UndP (red) could first bind at R290, near the cytoplasmic face of the inner membrane. (**b**) The substrate could then diffuse along a pathway lined with conserved hydrophobic (teal) and positively charged (blue) residues. (**c**) Finally, the phosphate headgroup is coordinated by R122 and R200 at the acceptor site, where catalysis occurs. (**d**) Mutation of hydrophobic and positively charged conserved residues lining the region between JM1 and JM2 abrogates GtrB activity. Error bars are provided as s.e.m., *n*=3. (**e**) The architecture of GtrB may be shared by the DPMS complex. CDG1e mutations G152V and S248P in the conserved JM1 and JM2 linkers are represented in purple and red, respectively. (**f**) Phenotypes typical of *dpm1* loss-of-function mutations are fully evident at 2 days post fertilization (d.p.f.) in the zebrafish embryo. Morphants show smaller head (microcephaly) and smaller eyes, kinked tail and occasional vascular defects in the tail vein (arrowhead). Scale bar, 500 μm (**g**) Functional analysis of *DPM1* mutants was performed by injecting human *DPM1* mRNA following *dpm1* morpholino injection and embryos were scored as normal or affected. Human *DPM1* mRNA was able to improve the morphant phenotypes (see Supplementary Fig. 8f for details) and the effect of different mutations was normalized to rescue levels. mRNA carrying the R147A or R234A mutations (equivalent to R122 and R200 in GtrB, respectively) showed almost complete loss of function. Known human missense changes (R92S, G152V and S248P) were also tested. R92S and G152V greatly abolished protein function, whereas S248P, which leads to milder human phenotypes, showed substantial residual activity. The presumed location of G152V and S248P on the juxtamembrane region is shown. R92S is expected to reside close to the active site in the GT domain. Error bars: s.e.m., **P<0.01, ***P<0.001, unpaired Student’s *t*-test for each mutant, *n*=6 (R147A), *n*=6 (R234A), *n*=3 (R92S), *n*=5 (G152V), *n*=4 (S248P). NS, not significant.

The active site of GtrB_*Syn*_ is located in the cytosol ∼15 Å from the membrane (Supplementary Fig. 11). This raises the question of how the hydrophobic acceptor from the membrane and hydrophilic donor from the cytosol are brought into proximity for catalysis to occur; that is, whether the GT domain moves to the membrane, or whether the lipid-phosphate acceptor is translocated to the active site. We favour the latter mechanism for three reasons. First, ∼900 Å^2^ of predominantly hydrophobic surface is buried between each pair of GT domains in the cytosol, with eight inter-subunit hydrogen bonds and two salt bridges formed at each interface (Supplementary Fig. 2), suggesting a stable structure that is unlikely to disassemble for the GT domain to move to the membrane. Second, the enzyme is highly specific for acceptors built from isoprenyl units (Supplementary Fig. 10), but the lack of strong conservation in the TM region (Supplementary Fig. 11) suggests that specificity for the lipid may reside elsewhere in the protein. Third, we have identified several absolutely conserved hydrophobic and basic residues in the juxtamembrane region (Supplementary Figs 11 and 12a) that could in principle form a solvent-shielded pathway for translocation of substrate from the membrane to the active site where the acceptor phosphate is bound by the side chains of R122 and R200 (Fig. 4a–c). Functional requirements for these residues (Fig. 4d), and their conserved pattern (Supplementary Figs 11 and 12a), lead us to put forth the hypothesis that as the UndP diffuses along this pathway, with its isoprenyl groups shielded by the hydrophobic residues, the phosphate headgroup could form transient ionic interactions with the positively charged moieties of the basic residues (Fig. 4a–c). To investigate this hypothesis, we first produced GtrB_*Syn*_ with mutations at each of the conserved residues R122, R125, K132, A136, Y140, R200, L212, A216 and R290, in the juxtamembrane region. Mutations at any of these sites led a complete loss of enzymatic activity (Fig. 4d). However, the mutant proteins could be expressed, solubilized and purified in a non-ionic detergent at levels comparable to wild type, with a similar elution profile in size-exclusion chromatography, suggesting that the overall structure was unaffected (Supplementary Fig. 13). As further proof that the fold of GtrB_*Syn*_ was unaffected by mutagenesis, we crystallized and determined the structure of several of these point mutants (Y209A, L212F, A216M, A136M, R122Q, T135A, R145A). Finally, functional assays on single-point mutations of each of the residues of JM1 and JM2 to alanine show that the entire region is extremely sensitive to even minor perturbations in structure (Supplementary Fig. 14), highlighting the importance of the juxtamembrane region in GtrB function.

### GtrB as a structural model to study DPMS function

Although PI-GTs have high sequence variability, and they may consist of one or multiple chains, the overall architecture comprising a soluble GT domain, a juxtamembrane region composed of two amphipathic helices and one or more TM segments is conserved. The TM region of the human DPMS complex is composed of two separate polypeptide chains, but there is high conservation in the key functional residues in the juxtamembrane region we have identified here (Supplementary Fig. 12a). Interestingly, disease-causing mutations G152V and S248P in human DPM1, the catalytic subunit of the DPMS complex, have been mapped to the JM1-GT and JM2-GT linkers, and are conserved in GtrB (Fig. 4e and Supplementary Fig. 12b)^26^,^27^. Another disease mutant, R92S, which causes the most severe phenotype, is expected to be located close to the active site. Together, these observations lead us to suggest that DPMS and other PI-GTs are likely to function by similar mechanisms. To investigate this hypothesis, we performed assays to determine whether GtrB and DPMS shared functional similarities. First, we asked whether GtrB could attach its donor substrate to DolP, the acceptor used by DPMS and all other eukaryotic PI-GTs. Indeed, GtrB_*Syn*_ could readily glucosylate DolP (Supplementary Fig. 12c). Second, we generated a zebrafish model of *dpm1* loss of function by knocking-down *dpm1* in zebrafish fertilized oocytes using two independent morpholino antisense oligonucleotides (MOs) targeting either translation (start site) or transcription (donor splice site in exon 1) at 4 and 8 ng, respectively. MO concentrations were carefully titrated to avoid nonspecific binding effects observed with high MO concentration, and knockdown was measured by quantitative PCR (Supplementary Fig. 15a,b). A scrambled control MO was used at equal concentrations to show that the effects were specific to *dpm1* (Supplementary Fig. 15c). Identical phenotypes were observed for both MOs and *dpm1* morphants recapitulated most features of congenital disorder of glycosylation type 1e (CDG1e) associated with *DPM1* mutations, including developmental delay, microcephaly, ocular defects and vascular anomalies, which were fully evident at 2–3 days post fertilization (d.p.f.; Fig. 4f and Supplementary Fig. 15d,e)^9^. Injection of human *DPM1* mRNA was able to significantly rescue the *dpm1* morphant phenotypes showing that the function is conserved between humans and zebrafish, and that the phenotypes observed are due to the loss of *dpm1* (Supplementary Fig. 15f). Injection of *DPM1* mRNA alone had no effect on development (Supplementary Fig. 15g). To test whether the proposed mechanisms of action is plausible in human DPM1, we co-injected MO with mRNA carrying mutations in R147 and R234, which correspond to R122 and R200 in GtrB_*Syn*_. The mutant mRNAs failed to rescue *dpm1* knockdown, suggesting that these residues are critical for function (Fig. 4g). Finally, we tested all known human mutations, R92S, G152V and S248P, which lead to variable phenotypic presentations. As observed for wild-type DPM1, overexpression of mutated mRNA alone had no effect indicating that these missense changes do not generate a gain of function (Supplementary Fig. 15g). R92S and G152V, which cause severe CDG1e, showed severe loss of function, as was previously shown in enzymatic assays^9^,^26^, whereas S248P, which causes a milder version of the disease^27^, only partially affected DPM1 function (Fig. 4g). Interestingly, a similar trend in activity levels was observed in GtrB_*Syn*_ for the G127V (inactive) and T205P (partially inactive) mutants (Supplementary Fig. 12d).

## Discussion

The structure of GtrB from *Synechocystis* sp. determined here at 3.0 Å resolution, is the first for a representative PI-GT, enzymes that attach a sugar to a lipid carrier for use in glycosylation reactions^3^. GtrBs, including GtrB_*Syn*_, attach a glucose molecule to UndP^11^,^12^, the conserved sugar-carrier lipid in prokaryotes (Fig. 1). The structure shows that GtrB is a tetramer in which each protomer has two membrane-spanning helices with a catalytic domain similar to those of soluble GTs attached through a juxtamembrane region (Fig. 2). Although the catalytic GT domain and the JM region are conserved across the family, it remains unclear whether other PI-GTs will have similar TM topologies.

Similarities in the catalytic domain with other GT-A fold GTs, coupled to functional mutagenesis data on GtrB_*Syn*_, have allowed us to identify a conserved aspartic acid (D157) as putative catalytic acid (Fig. 3b), and propose a scheme for how the reaction catalysed by GtrB may take place (Fig. 3c).

In the GtrB_*Syn*_ structure, the GT domains of the tetramer are ∼15 Å distant from the membrane surface (Supplementary Fig. 11), and separated from it by the juxtamembrane region. This arrangement raises the question of how hydrophobic substrates originating from the membrane and hydrophilic substrates from the cytosol are brought into apposition for catalysis. Overall our results, including the apparent rigidity of the assembly between the four catalytic domains of the tetramer (Supplementary Fig. 2) and functional loss by mutations in the juxtamembrane region (Fig. 4d and Supplementary Fig. 14), favour a mechanism where hydrophobic polyisoprenyl-phosphates translocate from the membrane to the cytosolic-active site (Fig. 4a–c), although further studies will be required to understand the molecular details of this mechanism.

The sequence conservation we observe suggests that all PI-GTs are likely to function similarly. The GtrB structure, together with results of functional mutagenesis on this enzyme and on the human DPM1 in a zebrafish model, also presented here (Fig. 4e–g and Supplementary Fig. 14), provide a framework for understanding PI-GT structure and function in both prokaryotes and eukaryotes.

## Methods

### Target identification and mutagenesis

GtrB from the cyanobacterium *Synechocystis* sp. PCC 6803 (UniProt, accession # Q55487) was initially identified as a promising candidate for crystallization experiments by the NYCOMPS (New York Consortium on Membrane Protein Structure) high-throughput target identification and screening pipeline^28^,^29^. The open reading frame encoding GtrB was amplified by PCR using *Synechocystis* sp. PCC 6803 genomic DNA as template. Primers used for amplification were 5′-tacttccaatccaatgccACCATTGAACTGTCTATTGTGATT-3′ (forward) and 5′-ttatccacttccaatgCTAATTAAGTTTTTCCAATGGCAAA-3′ (reverse), where upper case letters indicate gene-specific sequences and lower case letters indicate vector-specific sequences. The amplified product was cloned into the expression vector pMCSG7 by ligation-independent cloning, introducing a hexahistidine tag and a tobacco etch virus (TEV) protease cleavage site at the N-terminus of the polypeptide^30^. The identity of the resulting construct was verified by DNA sequencing. Sequence alignments were performed using PRALINE and PROMALS-3D (refs 31, 32). All point mutants were prepared using the QuikChange site-directed mutagenesis kit (Agilent).

### Protein expression and purification

To produce both native and the selenomethionine-substituted protein, GtrB_*Syn*_ was overexpressed in *E. coli* strain BL21 (DE3) pLysS. Cells were grown in 2xYT medium containing 100 μg ml^−1^ ampicillin and 50 μg ml^−1^ chloramphenicol at 37 °C. At an OD_600_ value of 0.8, temperature was reduced to 22 °C and protein expression induced 15 min later with the addition of 0.2 mM isopropyl β-D-thiogalactopyranoside. When producing the selenomethionine-substituted protein, the protocol was the same aside from the following. Cells were grown in minimal media containing M9 salts (M9 SeMET High-Yield Growth Media Kit, Shanghai Medicilon Inc.) with a supplemental 150 mg of selenomethionine per litre of culture, and protein expression was induced with 0.4 mM isopropyl β-D-thiogalactopyranoside. The purification protocol, as described below, is the same for both native and the selenomethionine-substituted protein. Cells were harvested after overnight induction, then re-suspended in lysis buffer (20 mM Na-HEPES, pH 7.5, 150 mM NaCl, 20 mM MgSO_4_, 10 μg ml^−1^ DNase I, 10 μg ml^−1^ RNase A, 1 mM tris(2-carboxyethyl)phosphine (TCEP), 1 mM phenylmethanesulfonylfluoride and Complete Mini EDTA-free protease inhibitor cocktail (Roche) as described in the instructions. Cells were lysed using an Avestin Emulsiflex C3 homogenizer, and then solubilized for 1 h by addition of (Anagrade, Affymetrix) to 1% (w/v) in a typical volume of 50 ml for a pellet from an 800 ml growth culture. After pelleting insoluble debris by ultracentrifugation (134,000*g*, 30 min) and filtering the supernatant (0.22 μm), solubilized GtrB_*Syn*_ was purified by metal-affinity followed by size-exclusion chromatography (SEC) using a 24-ml Superose 12 column (GE Healthcare). The SEC buffer contained 20 mM HEPES, pH 7.0, 150 mM NaCl, 1 mM TCEP and 0.2% (w/v) DM. Before crystallization, the peak fractions from SEC were concentrated typically to 15 mg ml^−1^ (estimated from *A*_280 nm_) using a centrifugal concentrator (Millipore) with a molecular weight cutoff of 50 kDa. Approximately 2 mg of purified GtrB_*Syn*_ could be obtained starting from an 800-ml bacterial culture. Small-scale expression tests were performed by essentially the same protocol as for the larger-scale purification, starting from 80 mg of a cell pellet taken from a larger-scale protocol. For small-scale expression tests, cells were lysed by sonication.

### Crystallization

Initial crystals were obtained by sitting drop vapour diffusion at 22 °C. The optimized crystallization protocol involved dispensing 600 nl of the purified protein solution (15 mg ml^−1^) and 300 nl of reservoir solution (containing 16-18% PEG600 (v/v), 0.12 M Tris/HCl, pH 9.0, 0.1 M NaCl and 1 mM TCEP) under 1 μl of silicone oil, in 96-well Axygen sitting-drop plates. Crystals typically appeared after 1–2 days and grew to a final size of ∼150 μm in each dimension. Before cryoprotection, a reservoir solution containing 5 mM MnCl_2_ and 25 mM UDP-glucose was added to the crystal-containing drop for 1 h. The crystals used for the tungstate anomalous difference Fourier map were soaked overnight in reservoir solution containing 10 mM sodium tungstate before cryoprotection. Crystals were flash-frozen by immersion in liquid nitrogen after incubating briefly in reservoir solution containing 28% (v/v) PEG600. Crystals of the native and the selenomethionine-substituted protein typically diffracted anisotropically to 3.2–3.8 Å resolution. GtrB_*Syn*_ crystallizes in space group C2, with unit cell parameters (Å) *a*=157.3, *b*=137.5, *c*=101.4, *β*=98.5°. One tetramer is in the asymmetric unit. Crystals of the F215A mutant were produced in the same way and diffracted isotropically to 3.0 Å resolution.

### Diffraction data collection and processing

Diffraction data were collected on beamline X4C at the NSLS, Brookhaven National Laboratory, and on beamline 24-ID-C at the Advanced Photon Source. Data sets collected above the Se K-edge from four selenomethionine-substituted crystals were merged and used for structure solution at 3.2 Å by multi-crystal SAD^14^. The data were indexed, integrated, scaled and merged using XDS and XSCALE^33^. Selenium sites were located using SHELXD, and refined in SHARP^34^. Density modification, including solvent flattening, histogram matching and four-fold non-crystallographic symmetry averaging, was performed using DM to give an initial experimentally phased electron density map^35^.

### Model building and refinement

An initial model was built using the auto-tracing option of SHELXE, and improved using BUCCANEER^36^,^37^,^38^. This model was manually completed using COOT and refined using the PHENIX crystallographic software package, alternating between cycles of manual building in COOT and refinement in PHENIX^39^,^40^. Translation/Libration/Screw (TLS) parameterization was employed in the latter stages of refinement (using phenix.find_tls_groups to generate Translation/Libration/Screw (TLS) group boundaries), and riding hydrogens were included in the last cycle of refinement. Torsion-angle non-crystallographic symmetry restraints were used throughout refinement. The final models have an *R*/*R*_free_ of 0.2281/0.2595 (WT) and 0.2685/0.3044 (F215A). A snapshot of typical electron density in the final 2mF_o-_DF_c_ map is included as well as the anomalous difference map derived from selenomethionine-labelled wild-type crystals used for phasing and sequence registration (Supplementary Fig. 5). The quality of the final models was analysed using the validation module of the PHENIX package, which incorporates Molprobity clash score, density correlation and rotamer analysis^41^. For F215A, 93% of residues are in the favoured region of the Ramachandran plot, with 0.3% outliers. Full data collection and refinement statistics are given in Supplementary Table 1. Protein structure figures were prepared using UCSF Chimera^42^. Calculations of the surface area buried at the subunit interfaces were performed using the PISA interface in COOT^43^.

### Mass-spectrometry analysis of lipids

The procedures for lipid extraction and liquid chromatography-mass spectrometry analysis were previously described^44^. Briefly, lipids were extracted from whole-cell samples (of either GtrB_*Syn*_-overexpressing cells or a negative control expressing an unrelated membrane protein) using a neutral Bligh-Dyer method^45^. After extraction, the dried lipids were dissolved in chloroform/methanol (2:1(v/v)). The extracted lipid species were separated by normal phase liquid chromatography (LC) using an Ascentis Silica HPLC column. LC eluents were injected into the ion spray source of a TripleTOF 5,600 quadrupole time-of-flight tandem mass spectrometer (AB SCIEX. The mass spectrometry/mass spectrometry analysis used nitrogen as the collision gas. Data analysis was performed using the Analyst TF 1.5 software.

### *In vitro* functional assay of GtrB activity

An assay for the activity of wild-type and mutant GtrB_*Syn*_ constructs using UDP-^14^C-glucose as a radiolabelled tracer was adapted from a previously published protocol^11^. Briefly, crude membranes were isolated from cell lysate by centrifugation at 134,000*g* for 1 h and dispersed in resuspension buffer (100 mM Tris/acetate pH 8.5) before storage at −80 °C for later use. On the day of the assay, the membranes were thawed, resuspended by sonication and washed two times with resuspension buffer. One milligram of crude membranes was used for each reaction, in a final volume of 250 μl. 1 mM EDTA, 10 mM MgCl_2_ and a variable (0–200 μM final concentration) amount of UndP (Indofine Chemicals) were added to the resuspended membranes, followed by a sonication step to facilitate incorporation of the UndP into the membrane. The radiolabelled substrate (0.25 μM UDP-^14^C-glucose) was then added and the mixture incubated at room temperature for 1 h. After extracting twice with butanol followed by two washes with water, 180 μl of the organic phase was combined with 10 volumes of scintillation fluid and radioactivity measured using a scintillation counter (Perkin Elmer). Assays testing acceptor lipids other than UndP were performed in the same way, with 100 μM of the alternate lipid in place of UndP. DolP, decaprenyl phosphate, octaprenyl phosphate and hexaprenyl phosphate were obtained from Indofine Chemicals, and dodecyl phosphate and phosphatidic acid were obtained from Sigma-Aldrich.

Alternatively, functional assays were also performed using purified protein reconstituted into liposomes. Liposomes and proteoliposomes were prepared as described^46^. Briefly, *E. coli* polar lipid extract (Avanti) and phosphatdiylcholine (PC, Avanti) were mixed in a 3:1 ratio (w/w) by dissolving in chloroform, and chloroform was removed under a stream of nitrogen gas. Lipids were resuspended in 100 mM HEPES, pH 7.5, buffer containing 1.5% (w/v) 1-O-n-Octyl-β-D-glucopyranoside (OG, Anagrade, Affymetrix) and the detergent was removed by dialysis. The resulting liposomes were divided into aliquotes, frozen in liquid nitrogen and stored at −80 °C. For proteoliposome formation, the concentration of thawed liposomes was adjusted to 10 mg ml^−1^ and 0.11% (w/v) Triton X-100 was added to the liposome-containing solution and purified protein was then added in a ratio of 1:80 (0.125 mg protein to 10 mg lipid), and after a 15-min incubation, detergent was removed by the addition of Bio-Beads SM-2 (Bio-Rad). Proteoliposomes were separated and removed from the Bio-Beads by careful pipetting, their concentration adjusted by ultracentrifugation and resuspension in the correct volume of buffer. Proteoliposomes were divided into aliquots, flash frozen in liquid nitrogen and stored at −80 °C. 5 μg of proteoliposomes at 10 μg ml^−1^ were used per assay, in a final volume of 250 μl. All subsequent steps were performed as described above for functional assays performed on membranes. Assays using protoliposomes were used to test activity of G127V and T205P GtrB_*Syn*_ mutants.

Protein expression levels were normalized by quantifying the intensity of bands on an SDS–PAGE gel, after solubilization and purification by metal-affinity chromatography in DM, using ImageJ^47^. The expression levels of mutants were compared with wild-type protein, and activity levels were then adjusted based on this normalization.

### Functional assay of human DPM1 activity in zebrafish

Fertilized zebrafish (*Danio rerio*) oocytes were obtained from Oregon AB breeders maintained in the Animal Research Facility at the George Washington University as described earlier^48^. All animal work was performed in compliance with protocols approved by the George Washington University Animal Care and Use Committees. Microinjection was performed on a PLI-100A Pico-Injector (Harvard Apparatus) at the one or two cell stage (20–45 min post fertilization) using pulled borosilicate pipettes. MO sequences were designed by Gene Tools LLC to target the start site or intron 1 splice donor site of *dpm1* (ENSDART00000079591) as follows: dpm1 start 5′-TACTTCTGCGGCTCGCCATGTTGGC-3′ and dpm1 splice 5′-TTCAGGTTTCGCAATTACCTTTCGC-3′. Standard control MOs were provided by Gene Tools. MOs were injected at 1–4 ng for dpm1 start or 8–20 ng for dpm1 splice in 1 × Danieau solution (58 mM NaCl, 0.7 mM KCl, 0.4 mM MgSO4, 0.6 mM Ca(NO_3_)_2_, 5 mM HEPES, pH 7.6) with 0.1% phenol red as injection dye. Full-length or mutated *DPM1* mRNA was cloned into pCS2+ for capped mRNA production. mRNA was produced using the Ambion mMessage mMachine SP6 trascription kit. Rescue injections were performed sequentially by injecting the MO and the mRNA independently at the 1–2 cell stage in 60–100 embryos per condition. For each clutch, at least four conditions were tested at the same time: un-injected wild types, MO injection, rescue (MO+wild-type mRNA), mutant (MO+mutated mRNA). Whenever sufficient embryos were available, an mRNA alone control was added or multiple mutants were tested in parallel. Embryos were maintained at 28.5 °C and staged at hours (h) and days (d) post fertilization. For imaging and analysis 2–3 d.p.f. embryos were anaesthetized using 0.4% Tricaine stock and imaged on a Leica M165 stereomicroscope. For knockdown quantification, mRNA was extracted from control and experimental embryos using Promega ReliaPrep RNA miniprep system and reverse transcribed using Bio-Rad iScript cDNA Synthesis kit. qPCR was performed using Bio-Rad SsoFast EvaGreen Supermix on a Bio-Rad CFX384 platform in the GWU Biomarker Core. Expression was normalized to ribosomal protein L8 (*rpl8*). Primer sequences are available in Supplementary Table 2.

## Additional information

**Accession codes:** Coordinates and structure factors have been deposited in the Protein Data Bank under the accession code 5EKP (WT) and 5EKE (F215A).

**How to cite this article:** Ardiccioni, C. *et al.* Structure of the polyisoprenyl-phosphate glycosyltransferase GtrB and insights into the mechanism of catalysis. *Nat. Commun.* 7:10175 doi: 10.1038/ncomms10175 (2016).

## Supplementary Material

Supplementary Information Supplementary Figures 1-15, Supplementary Tables 1-2 and Supplementary References

## Figures and Tables

**Table 1:** Data collection and refinement statistics.

	SeMet-GtrB*	**GtrB**	**GtrB (F215A)**	**GtrB (WO**_**4**_)
Wavelength (Å)	0.979	0.979	0.979	1.02
Resolution range (Å)	46.79–3.21 (3.40–3.10)	19.99–3.194 (3.308–3.194)	40.79–3.0 (3.107–3.0)	37.82–4.498 (4.658–4.498)
Space group	C 2	C 2	C 2	C 2
*Unit cell*				
*a*	155.866	157.331	154.159	155.858
** ***b*	138.771	137.518	142.263	141.618
** ***c*	101.048	101.448	102.024	101.463
** ***β*	98.249	98.5	97.158	97.99
Total reflections	863,961 (105,225)	260,786 (24,889)	182,341 (17,880)	98,386 (9,396)
Unique reflections	38,430 (4,716)	35,029 (3,408)	43,375 (4,247)	13,024 (1,282)
Multiplicity	22.5 (22.3)	7.4 (7.3)	4.2 (4.2)	7.6 (7.3)
Completeness (%)	98.8 (99.3)	98.0 (97.0)	98.0 (98.0)	99.0 (99.0)
Mean *I*/sigma(*I*)	21.5 (1.2)	12.84 (1.32)	17.65 (0.99)	7.81 (2.32)
Wilson B-factor	105.4	125.7	112.6	195.8
*R*_merge_	0.123 (3.55)	0.089 (1.32)	0.045 (1.30)	0.180 (1.71)
CC1/2	0.997 (0.519)	0.999 (0.684)	0.997 (0.692)	0.998 (0.763)
*R*_work_	—	0.220 (0.321)	0.249 (0.389)	—
*R*_free_	—	0.279 (0.374)	0.276 (0.415)	—
Number of non-hydrogen atoms	—	9,254	9,243	—
Macromolecules	—	9,150	9,139	—
Ligands	—	104	104	—
RMS (bonds)	—	0.009	0.004	—
RMS (angles)	—	1.19	0.54	—
Ramachandran favoured (%)	—	90	94	—
Ramachandran outliers (%)	—	0.86	0.52	—
Clashscore	—	14.95	5.77	—

Statistics for the highest resolution shell are shown in parentheses.

^*^Friedel pairs (I^+^/I^−^) treated as separate reflections; RMS, Root mean square.
