# Supplementary material for: Structure of the polyisoprenyl-phosphate glycosyltransferase GtrB and insights into the mechanism of catalysis
Source: Nat Commun. 2016 Jan 5;7:10175. doi: 10.1038/ncomms10175 (PMC4728340; doi:10.1038/ncomms10175)
Supplement: Supplementary Information — Supplementary Figures 1-15, Supplementary Tables 1-2 and Supplementary References [file ncomms10175-s1.pdf]

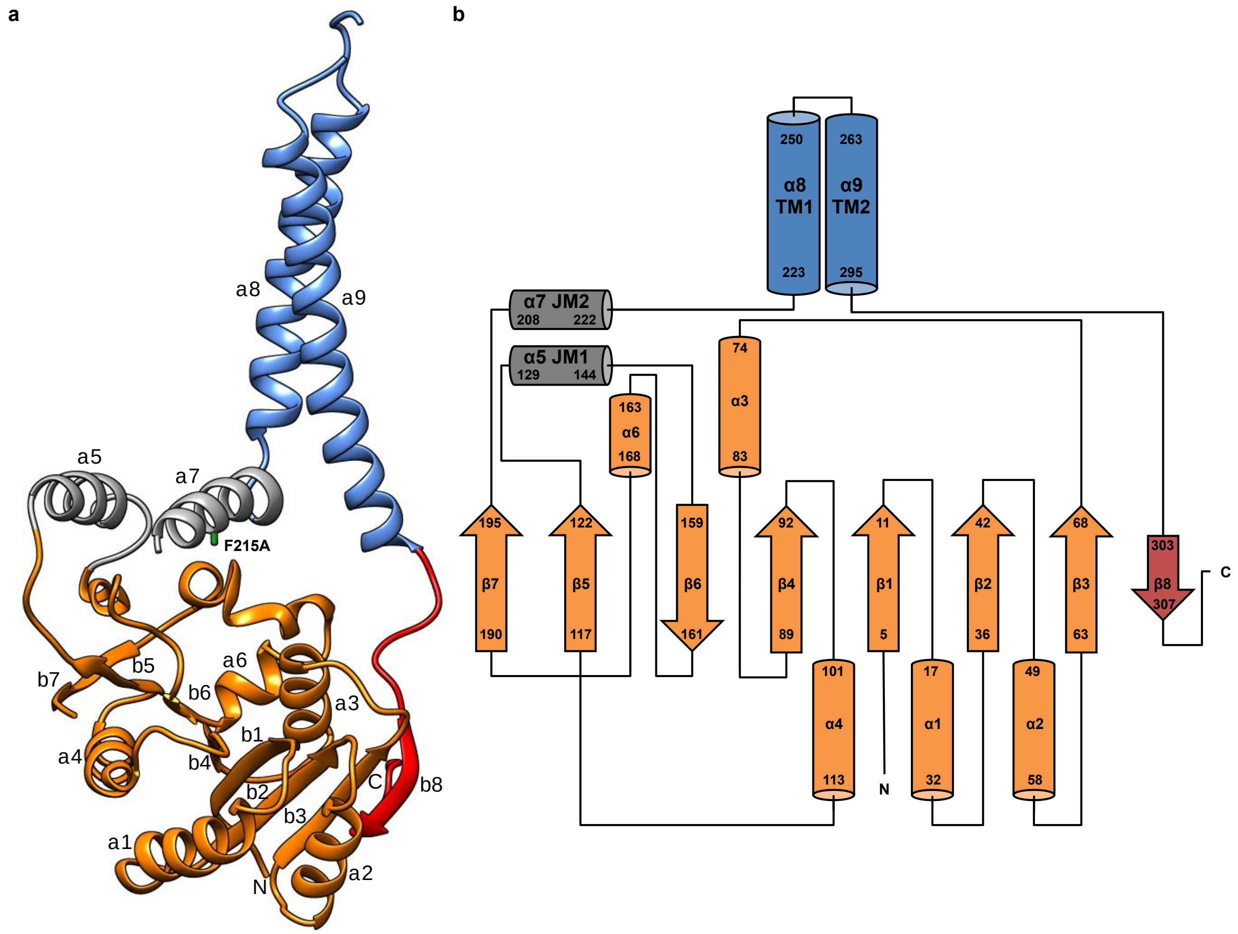

Supplementary Fig. 1 | Topology of GtrB. (a) A single subunit of the GtrB tetramer is shown, comprised of a GT-A fold GT domain (orange), two amphipathic JM helices (gray), two TM helices (blue), and a C-terminal β-hairpin (red). All α-helices and β-strands are labeled α and β respectively, and numbered progressively from the N to the C terminus, labeled N and C respectively. **(b)** The secondary structure and topology of GtrB, with β-strands depicted as arrows and α-helices represented as cylinders, numbered progressively, and with the number in sequence of the first and last residue of each element also shown. Secondary structural elements belonging to the GT domain are colored orange, the JM helices are colored gray, the TM helices are colored blue, and the C-terminal hairpin is colored red.

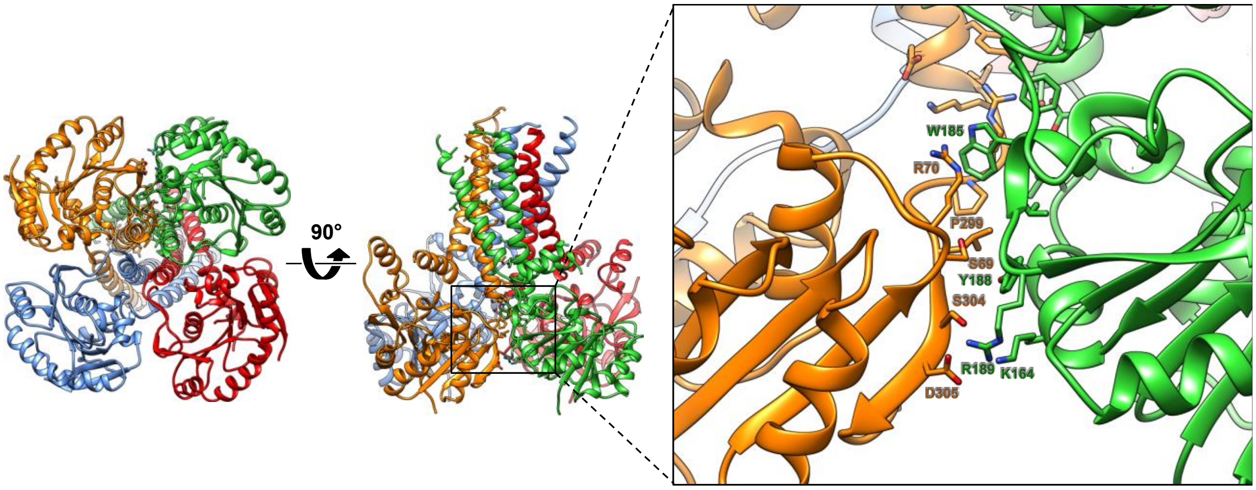

Supplementary Fig. 2 | The molecular interface between GT domains of GtrB protomers in the GtrB tetramer. The GtrB tetramer is shown in two views, from the cytoplasmic side (left) and from the membrane (right) which each protomer drawn in a different color. The inlet shows the dimer interface between GT domains, and residues involved are labeled.

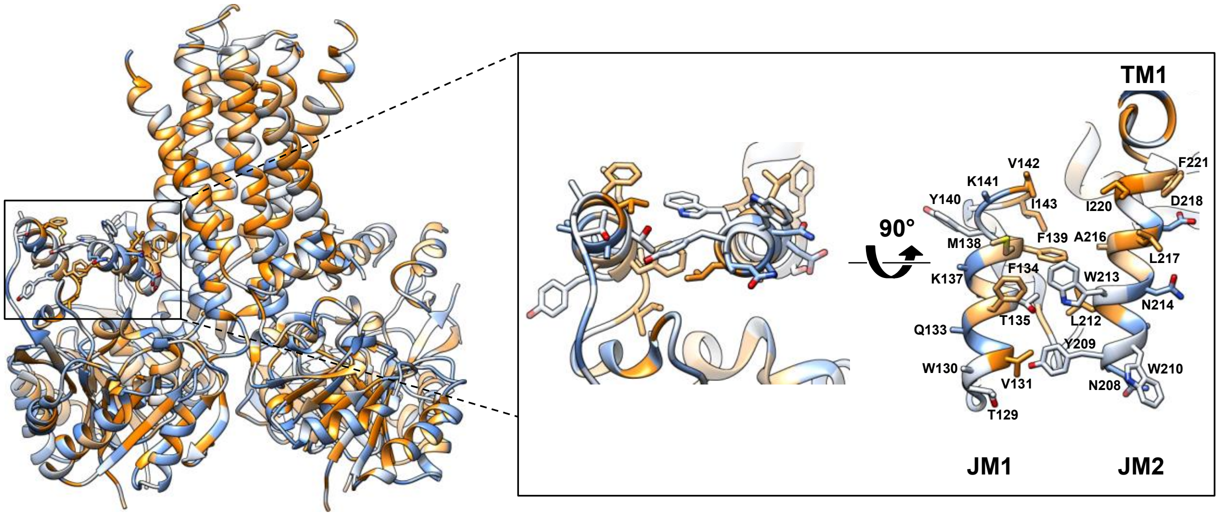

Supplementary Fig. 3 | The interface between JM helices in GtrB. The interface between the two JM helices is magnified in the inlet on the right, and shown in two orthogonal views. Residues are colored by the Kyte-Doolittle hydrophobicity scale[1] from orange (hydrophobic) to blue (most polar) and labeled.

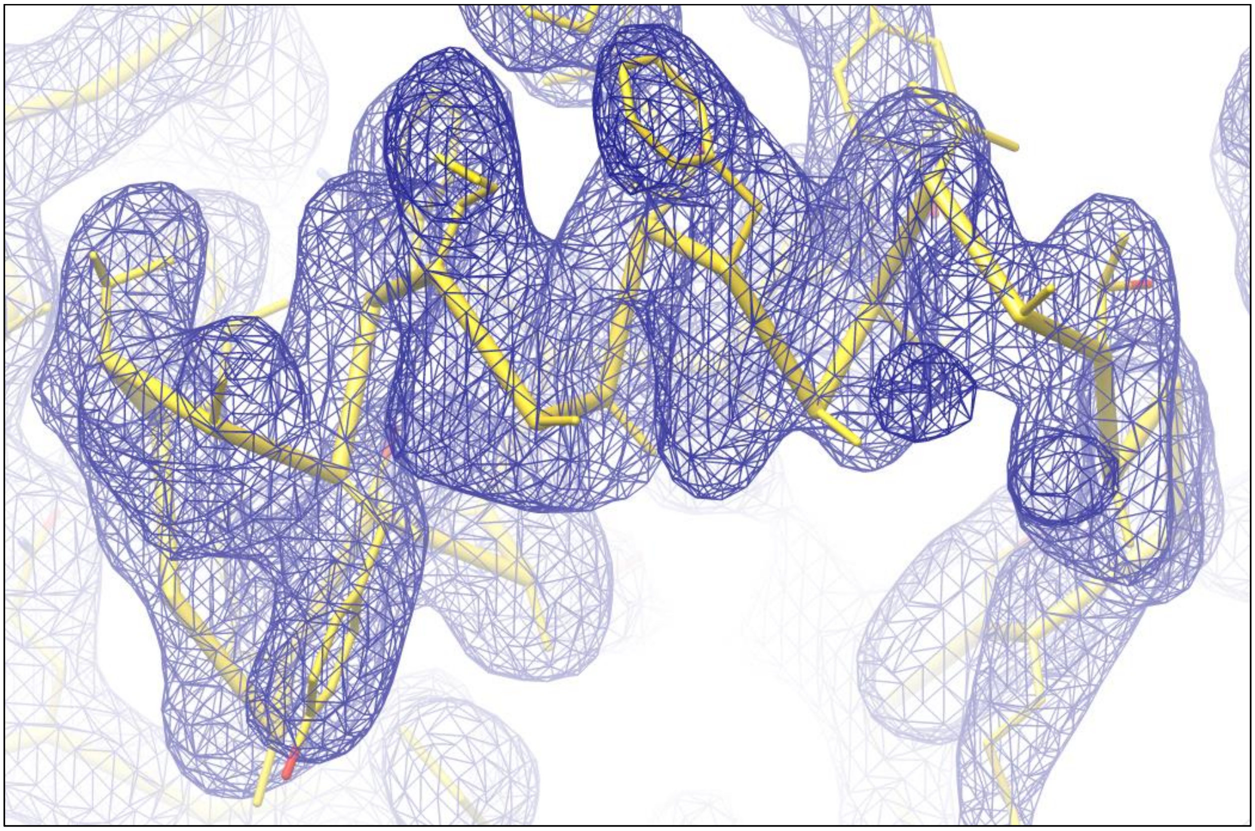

Supplementary Fig. 4 | Electron density in the juxtamembrane region. Final $2F_o$-F_c electron density map (blue mesh), in JM1/2 region (yellow Cα trace), contoured at 1 rmsd.

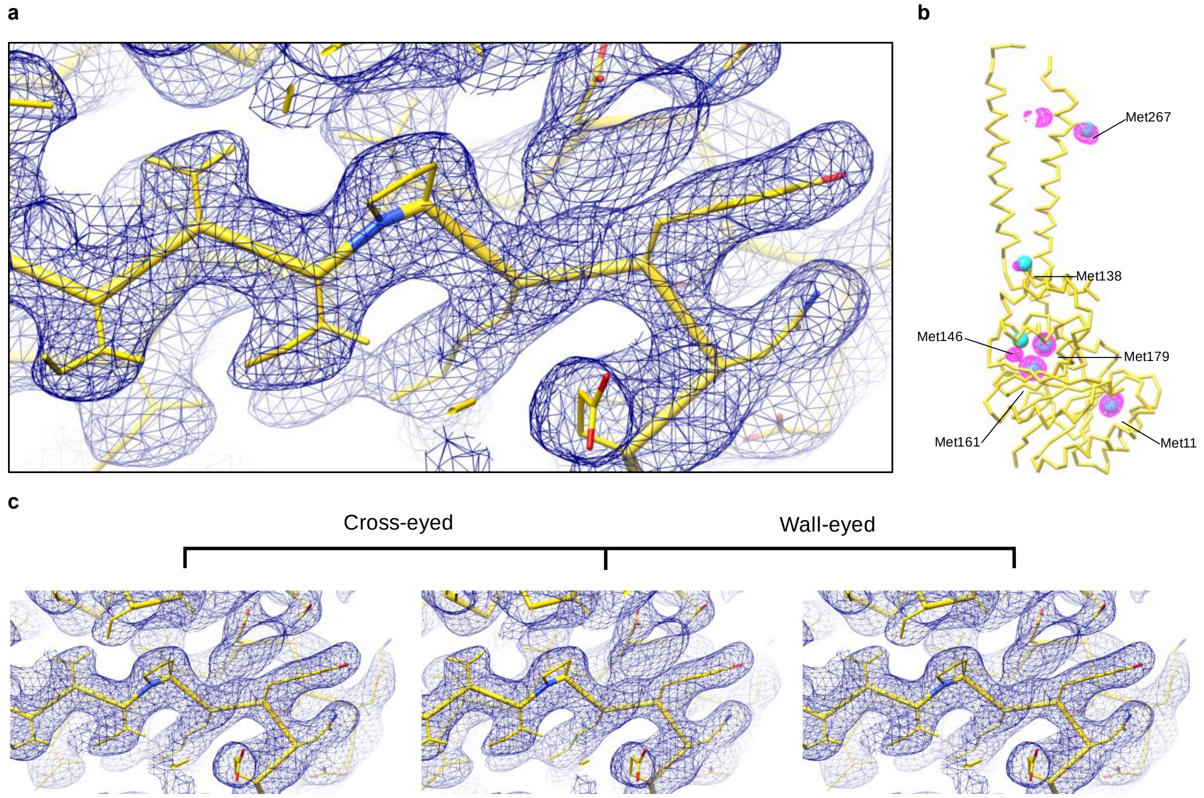

Supplementary Fig. 5 | Typical electron density and sequence assignment using selenomethionine markers. (a) Snapshot of electron density in the final $2F_o$-F_c map (sharpened with an applied B-factor of -50A^2), contoured at 1.2 rmsd, with the final refined model. (**b)** Cα-trace (yellow), of one protomer of the final refined model, with the Cα atoms of methionines represented as cyan spheres. An anomalous difference map, calculated from an isomorphous selenomethionine crystal and contoured at 3rmsd and displayed within 5Å of the model, is represented as purple mesh. A secondary peak is observed near M267, due to the proximity of this region to the equivalent residue in the neighboring protomer. (c) The same snapshot of electron density shown in (**a**), displayed here in cross-eyed (left) and wall-eyed (right) and stereo representation.

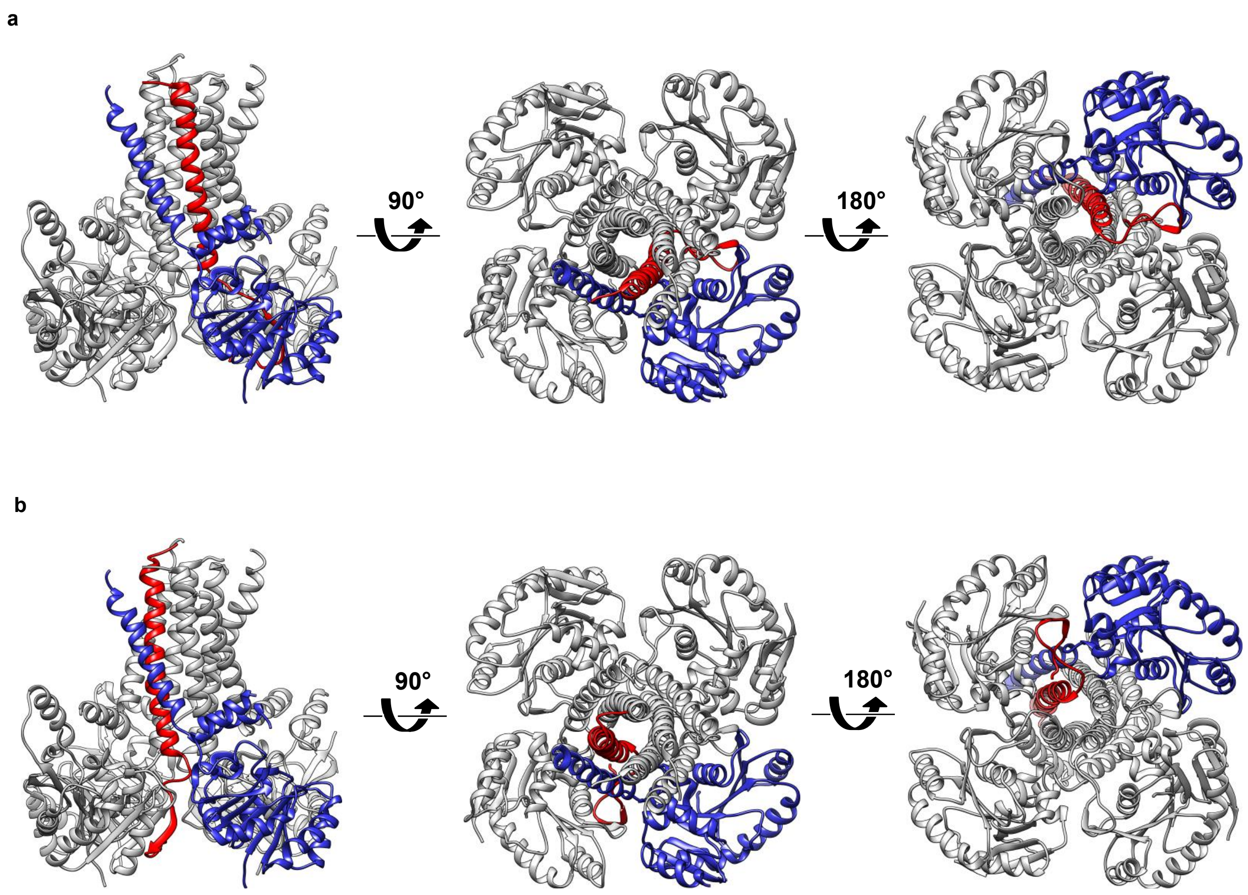

Supplementary Fig. 6 | Ambiguity in topology for GtrB. Two possible transmembrane topologies (**a, b**) for GtrB are represented in three different orientations - parallel to the membrane (left) from the cytosol (right) and from the extracellular compartment (center). The N-terminal and C-terminal portions of a single putative protomer are represented in blue and red respectively. The topology depicted in panel (a) is the one we deem most likely from our interpretation of the poor quality electron density map on the periplasmic side of the transmembrane helices, and has been used in all figures.

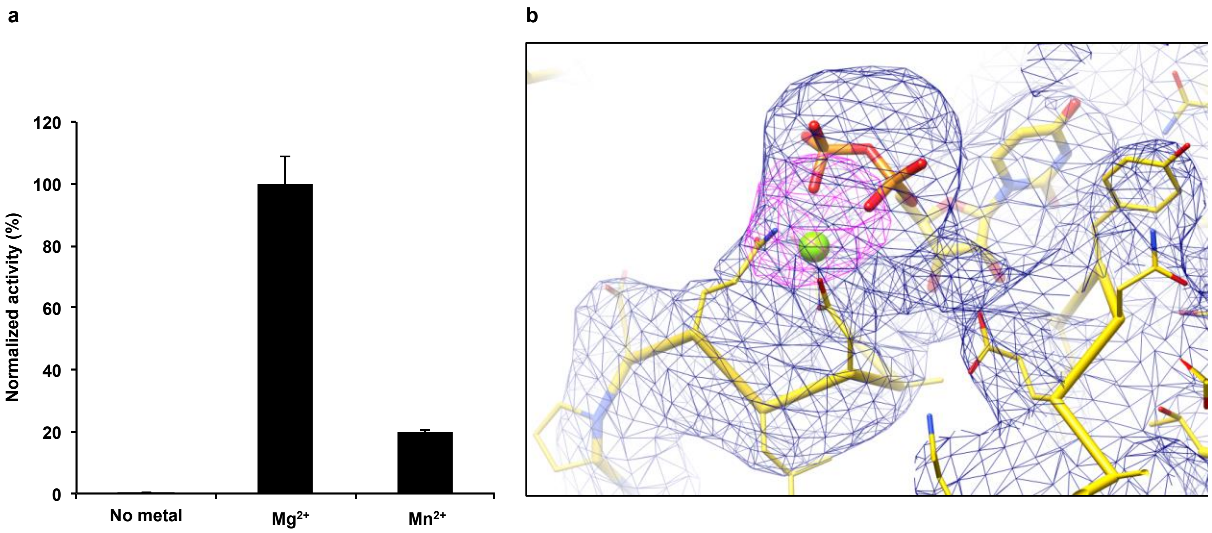

Supplementary Fig. 7 | Mn/Mg activity, Mn/UDP density. (a) GtrB requires divalent cations for activity, and is more active in the presence of magnesium than manganese ions. Error bars are represented as standard error of the mean, n=3. (**b)** Electron density at the UDP binding site. The final $2F_o$-F_c map is contoured at 1 rmsd and represented as blue mesh; An omit F_o-F_c map for the Mn-UDP is contoured at 4 rmsd and represented as green mesh; An anomalous difference Fourier map, calculated using reflections to a maximum resolution of 7 Å, is contoured at 4 rmsd and represented as purple mesh.

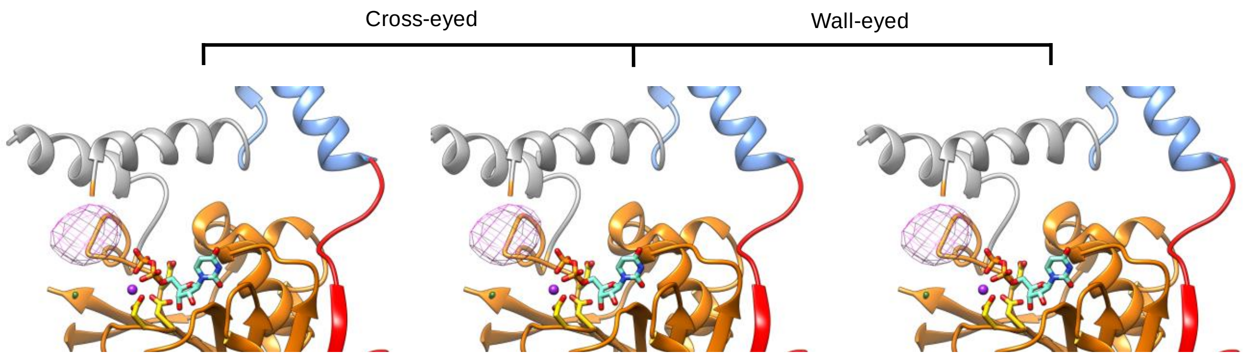

Supplementary Fig. 8 | Stereo representation of the active site region of GtrB. Wall-eyed stereo representation of the active site, in the GT domain (orange). The peak from an anomalous difference Fourier map (contoured at 5 σ above the mean), calculated from a tungstate-soaked wild-type crystal is represented as purple mesh. Residues R122 (green, Cβ only), D94, D96 and D157 (yellow) and the UDP are shown in stick representation. Mn^{2+} is represented as a purple sphere.

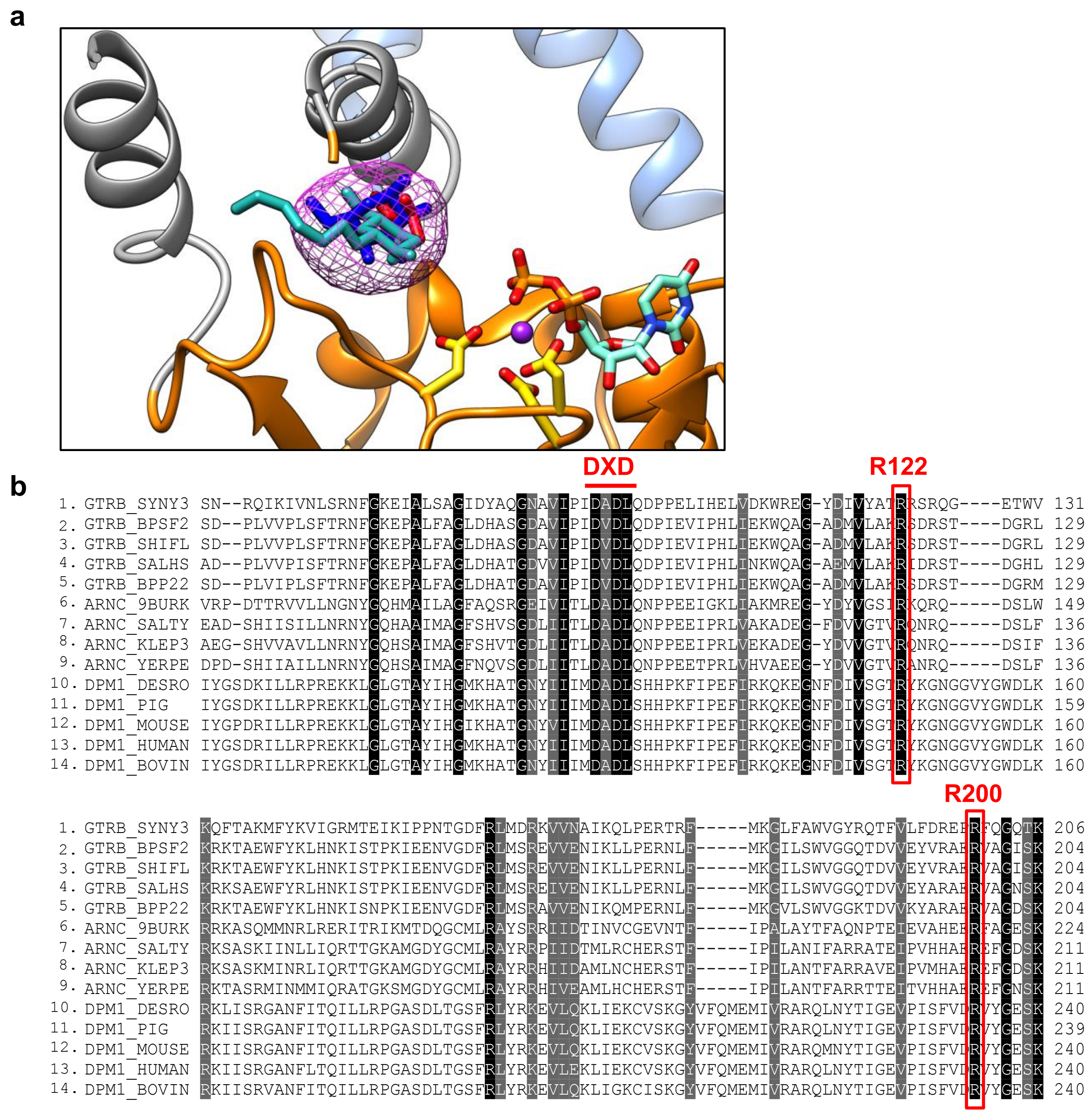

Supplementary Fig. 9 | Structural alignment of acceptor complexes, sequence alignment showing conservation of GtrB R122 and R200. (a) The position of bound tungstate (anomalous difference Fourier map, purple mesh, contoured at 5 rmsd) overlaid with acceptor positions from other GT acceptor complex structures (Blue, PDB ID 1FGG; Cyan, PDB ID 1R7T; Red, PDB ID 2Y4J). 1FGG is human glucuronyltransferase I in complex with the

trisaccharide GAL-GAL-XYL[2], rmsd 0.881 Å; 1R7T is human glycosyltransferase A in complex with 3-deoxy-acceptor analog inhibitor[3], rmsd 0.838 Å; 2Y4J is mannosylglycerate synthase from *Rhodothermus marinus* in complex with lactate[4], rmsd 1.154 Å. All are family 2 glycosyltransferases. (**b)** Two arginines near the acceptor site, R122 and R200 (red boxes) are conserved in GtrB, ArnC and DPM1. Sequences shown are of GtrBs (1-5), ArnC (6-9) and DPM1 (10-14), and are as follows: 1. Q55487; *Synechocystis sp.* 2. P68668; *Shigella phage SfII*. 3. P68667; *Shigella flexneri*. 4. B4TDI3; *Salmonella heidelberg*. 5. P57022; *Enterobacteria phage P22*. 6. G7HHI5; *Burkholderia cenocepacia H111*. 7. O52324; *Salmonella typhimurium*. 8. B5XTK8; *Klebsiella pneumoniae*. 9. Q8ZDX7; *Yersinia pestis*. 10. K9IHA9; *Desmodus rotundus*. 11. A5GFZ5; *Sus scrofa*. 12. O70152; *Mus musculus*. 13. O60762; *Homo sapiens*. 14. Q1JQ93; *Bos taurus*.

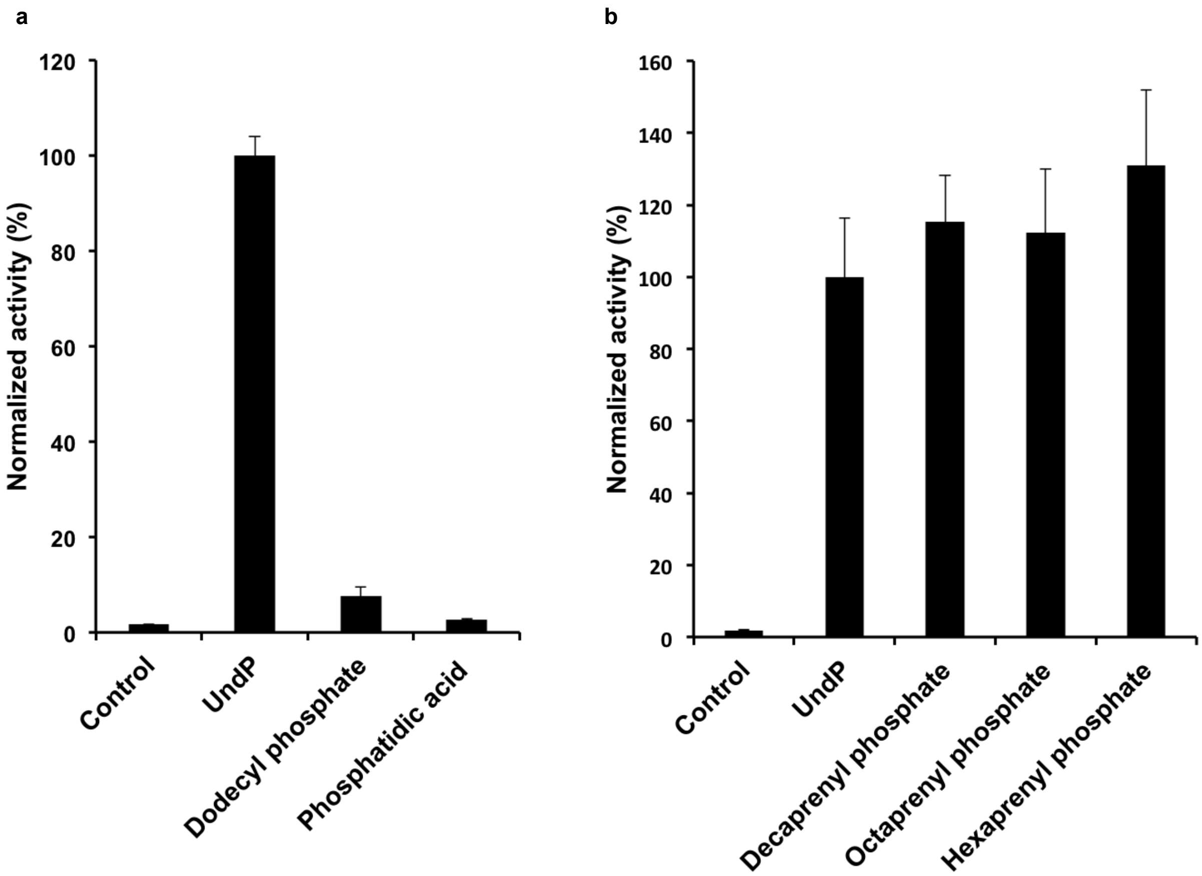

Supplementary Fig. 10 | Specificity of the isoprenyl units. (a) Activity of GtrB on UndP, dodecyl phosphate and phosphatidic acid (error bars depict S.E.M., n=3) (**b)** Activity of GtrB on UndP, decaprenyl phosphate, octaprenyl phosphate, and hexaprenyl phosphate (error bars depict S.E.M., n=3).

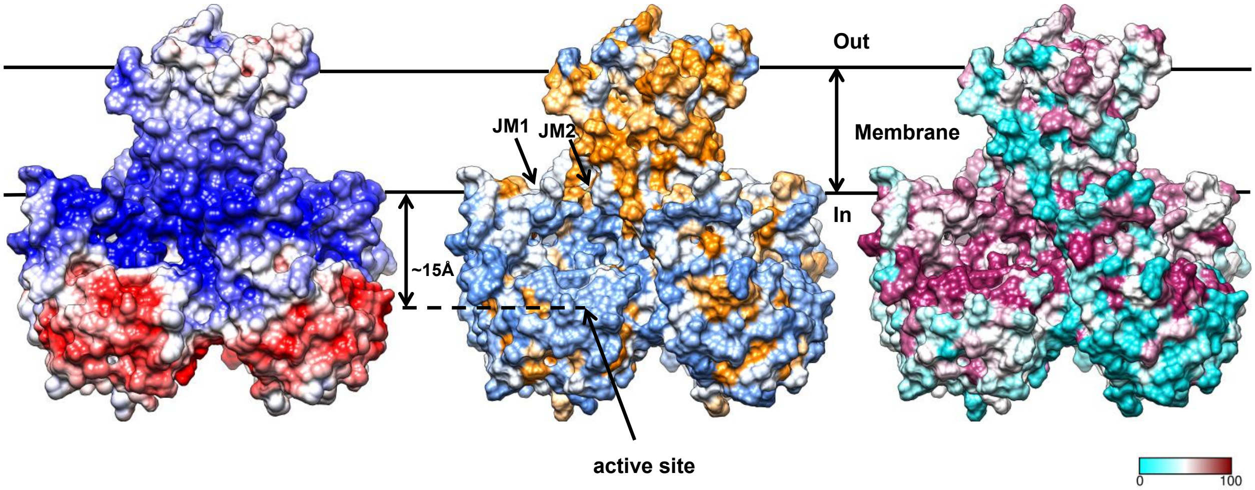

Supplementary Fig. 11 | Electrostatics, hydrophobicity and conservation of GtrB. GtrB is represented as an electrostatic surface potential (left), colored from -10 (red) to 10 (blue), as a molecular surface (centre) colored according to the Kyte-Doolitle hydrophobicity scale[1] from -4.5 (most polar, cornflower blue) to 4.5 (most hydrophobic, orange), and according to conservation (right) from maroon (absolutely conserved) to cyan (poorly conserved). The estimated position of the cytoplasmic and extracellular borders of the membrane are indicated (black lines). The positions of the two JM helices, and of the active site in the GT domain are indicated in the central image. The positions of the intracellular and extracellular borders of the membrane were calculated using the PPM server (http://opm.phar.umich.edu/server.php)[5], and the vertical distance between the predicted position of the intracellular surface of the membrane and the active site was measured using UCSF Chimera.

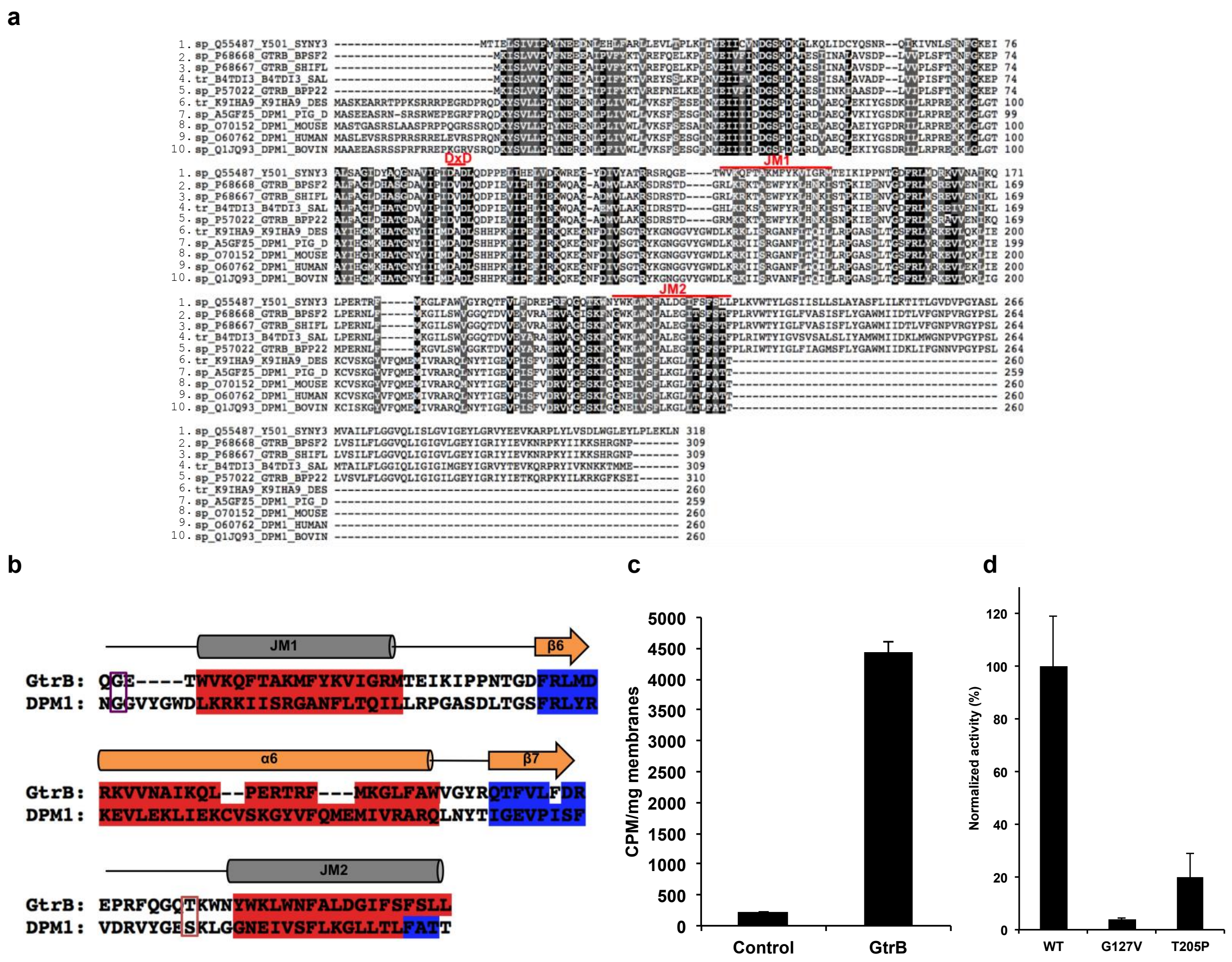

Supplementary Fig. 12 | Sequence alignment of DPM1 and GtrB sequences and DolP activity. (a) Multiple sequence alignment of GtrB and DPM1 sequences. The first five sequences are GtrB sequences from different species, the last five are DPM1 sequences. The top sequence is from *Synechocystis*. Sequences aligned using PROMALS-3D. Identical residues are highlighted in black, and similar residues in gray. JM1, JM2 and the DxD motif are indicated by red lines above the relevant regions of the sequence. Sequences shown are for GtrB (1-6) and DPM1 (7-10) and are as follows: 1. Q55487; *Synechocystis sp.* 2. P68668; *Shigella phage SfII*. 3. P68667; *Shigella flexneri*. 4. B4TDI3; *Salmonella heidelberg*. 5. P57022; *Enterobacteria phage P22*. 6. K9IHA9; *Desmodus rotundus*. 7. A5GFZ5; *Sus scrofa*. 8. O70152; *Mus musculus*. 9. O60762; *Homo sapiens*. 10. Q1JQ93; *Bos taurus*.

(b) Sequence alignment in the JM1/2 region of GtrB and human DPM1. Predicted secondary structure (PSIPRED) is highlighted in red (helices) and blue (β-strands). Mutations present in patients with congenital glycosylation defects G152V and S248P are boxed in purple and red, respectively. (**c**) Activity of GtrB on DolP (error bars depict S.E.M., n=3). Membranes expressing GtrB and a control protein were incubated with UDP-^{14}C-glucose and Mg^{+2} after sonication with 100 μM DolP, and the resulting ^{14}C-labeled product extracted in an organic phase prior to quantitation. Activity with this substrate is about 1/3 with respect to UndP at the same concentration. **(d)** Activity of G127V and T205P (G152V and S248P in DPM1, respectively) compared to the wild type $GtrB_{Syn}$. G127V is inactive and T205P shows a substantial (20% of WT levels) decrease in activity (error bars depict S.E.M., n=3).

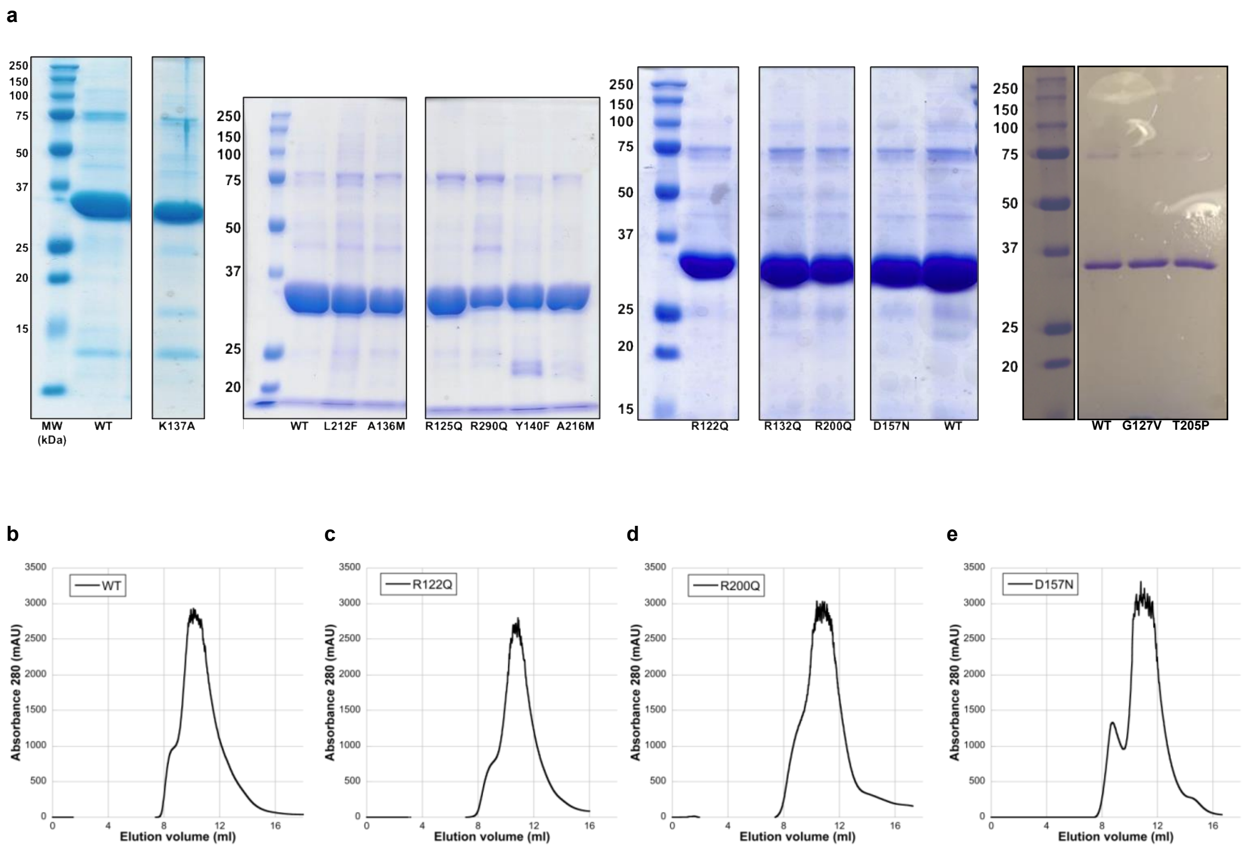

Supplementary Fig. 13 | Purification yields and stability of mutants in a non-ionic detergent. Representative SDS-PAGE gels showing that the yields of wild-type and mutant proteins after over-expression in *E. coli*, extraction and purification in the non-ionic detergent dodecyl-β-maltopyranoside and purification by metal-affinity chromatography is comparable. The size (kDa) corresponding to each MW marker is labeled on the left side of each set of gels shown. The SEC elution profiles of the **(b)** WT and three representative mutants, R122Q **(c)**, R200Q **(d)** and D157N **(e)** are comparable, suggestive of comparable stability of these proteins.

a

Sample	Activity
WT	1.0 ± 0.15
Y209A	0.18 ± 0.027
W210A	0.16 ± 0.013
K211A	0.54 ± 0.024
L212A	0.097 ± 0.0044
W213A	0.087 ± 0.010
N214A	0.27 ± 0.027
F215A	0.19 ± 0.044
L217A	0.17 ± 0.064
D218A	0.40 ± 0.026
G219A	0.15 ± 0.013
I220A	0.13 ± 0.0089
F221A	37 ± 0.26
S222A	0.99 ± 0.032
F223A	1.4 ± 0.014
S224A	1.4 ± 0.026
L225A	3.3 ± 0.16
L226A	2.3 ± 0.036
W130A	0.71 ± 0.023
V131A	0.89 ± 0.039
K132A	0.12 ± 0.0096
Q133A	0.25 ± 0.062
F134A	0.48 ± 0.023
T135A	0.32 ± 0.031
K137A	0.43 ± 0.057
M138A	0.84 ± 0.057
F139A	0.13 ± 0.0032
Y140A	0.083 ± 0.0096
K141A	0.50 ± 0.020
V142A	0.40 ± 0.020
I143A	0.97 ± 0.054
G144A	1.4 ± 0.068
R145A	6.7 ± 0.20
M146A	0.82 ± 0.040

b

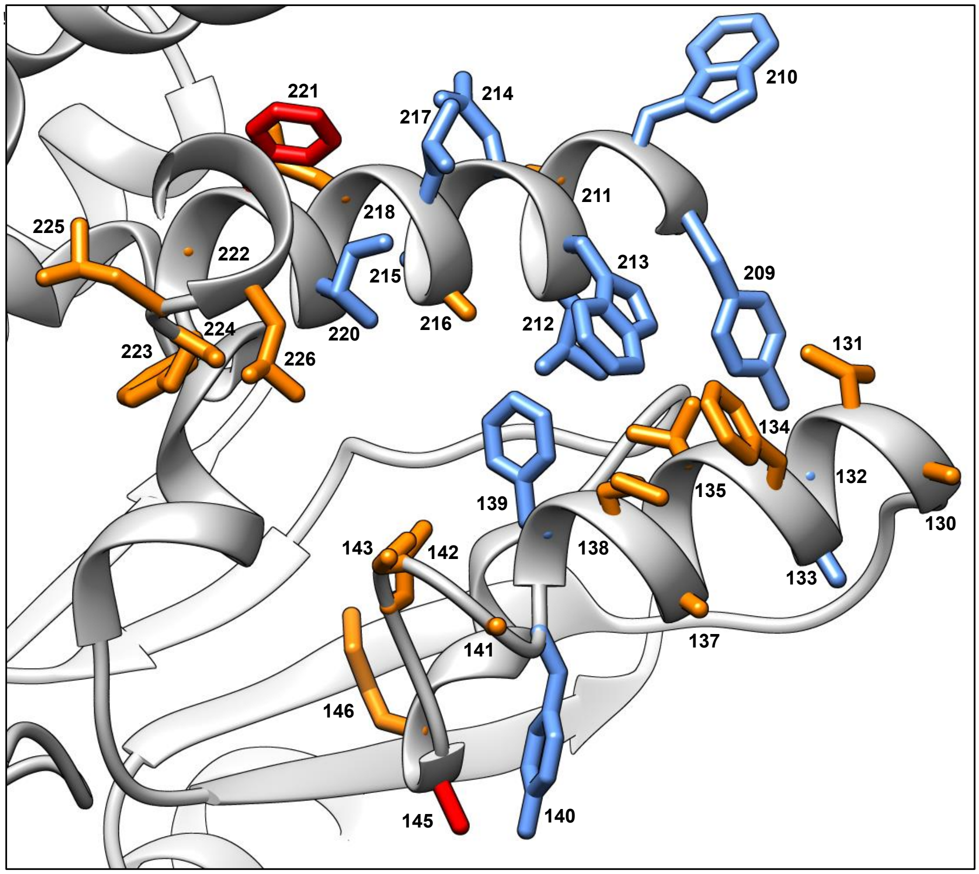

Supplementary Fig. 14 | Alanine scan of the JM region. (a) Activity and **(b)** structural representation of all the residues in the JM1 and JM2 mutated into alanine. Alanine mutants of residues colored in orange show an activity above 30% of WT levels, the blue ones below 30% and the once in red are more than twice as active as WT.

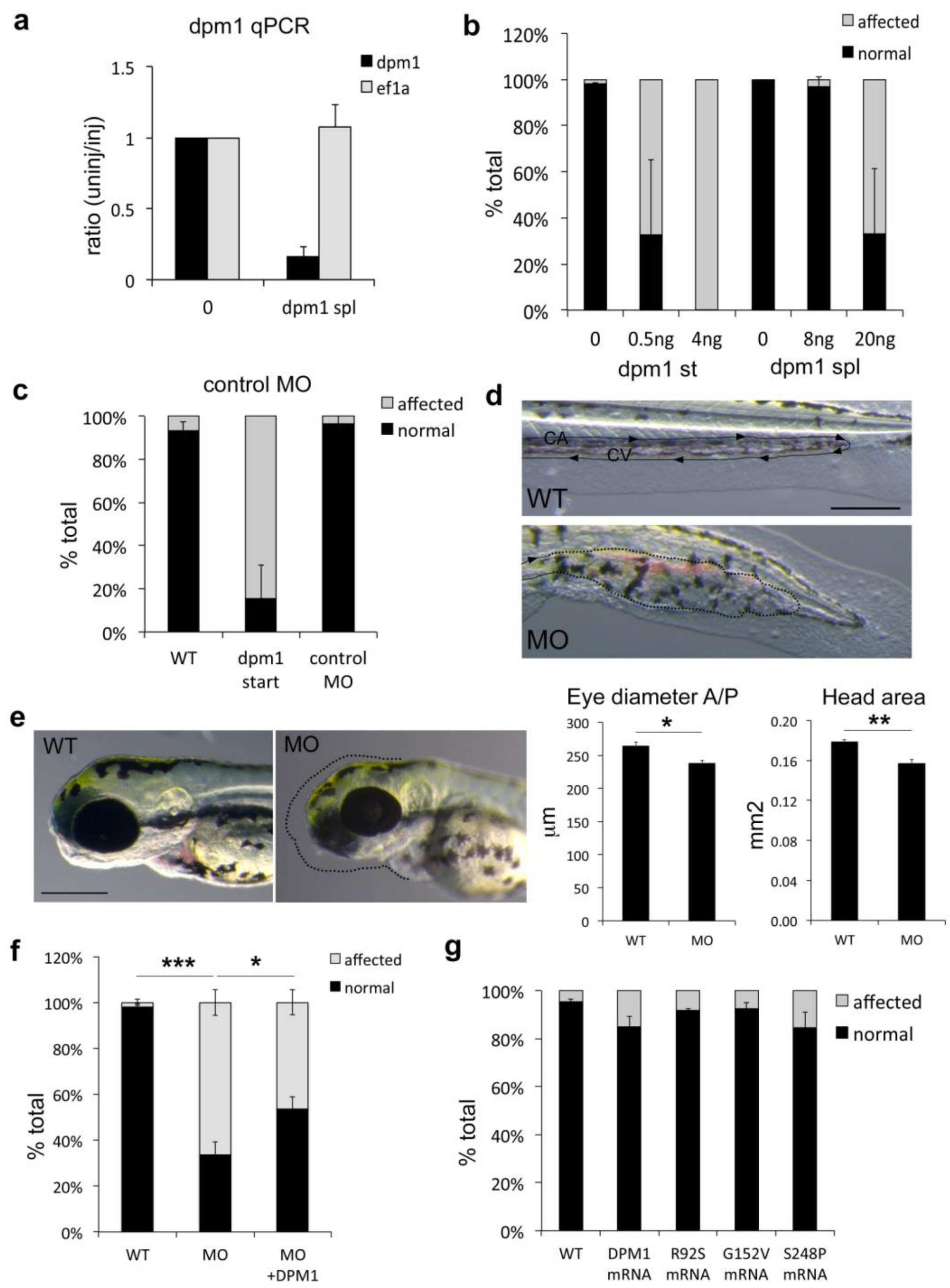

Supplementary Fig. 15 | *dpm1* knockdown in zebrafish recapitulates the human phenotypes caused by *DPM1* mutations. Multiple controls to confirm the effectiveness and specificity of the *dpm1* morpholinos (MOs) were performed. All error bars represent S.E.M. *=p<0.05, **=p<0.01, ***=p<0.001, unpaired student's t-test**,** N's are indicated for each experiment. **(a)**

Knockdown efficiency was measured in affected embryos using 10ng of *dpm1* splice MO at 1dpf. No difference is observed in expression of the housekeeping gene *ef1a*. (N=3) (**b**) Splice and start *dpm1* MOs caused identical phenotypes as shown in Fig. 4 and in this figure in **d** and **e** at different concentrations. As start MOs are usually more effective 4ng were sufficient to generate 100% affected embryos at 1dpf, while the splice MO needed to be injected at higher concentrations. (N=3) (**c**) Injection of a scrambled control morpholino in parallel to maximally effective concentrations of the start MO had no effect on the embryos, showing that these phenotypes are not due to injection and general MO toxicity. (N=4) (**d**) Vascular defects reminiscent of those observed in the patients can also be occasionally observed in the *dpm1* morphant. The caudal artery (CA) and caudal vein (CV) supply blood flow to the tail in the direction indicated by the arrows in the WT panel. In the morphants blood vessels appear fused and enlarged (outlined by the dotted line) leading to accumulation of blood in the distal portion of the tail. Scale bar: 200μm. (**e**) Microcephaly, a reduction in head size, and a reduction in eye size are observed in most morphants. Dotted lines in the morphants (MO) panel shows the outline of the wild-type (WT) head in black and of the WT eye in white for reference. Scale bar: 200μm. Quantification of the reduced eye diameter measured on the anterior/posterior (A/P) axis and of head area is shown (N=3). (**f)** Rescue experiments were performed using 2ng *dpm1* start MO injections as described in the Methods and 200pg of DPM1 mRNA, then the number of normal vs. affected embryos were tested. Despite embryos and larvae in the rescue condition appeared less affected than MO injected clutchmates, this difference was not clearly quantifiable due to the multiple phenotypes observed, so the total number of normal vs. affected was used (N=6). (**g)** To rule out possible gain of function effects of human missense mutations, normal and mutated DPM1 mRNAs were injected in isolation and none caused substantial defects (N=3).

Sample	Normalized Activity	Location
WT	1.0 ± 0.15	
R122Q	0.016 ± 0.0019	β5
R125Q	0.043 ± 0.0059	β5-α5 loop
G127V	0.040 ± 0.0036	β5-α5 loop
W130A	0.71 ± 0.023	α5 (JM1)
V131A	0.89 ± 0.039	α5 (JM1)
K132A	0.12 ± 0.0096	α5 (JM1)
K132Q	0.037 ± 0.0029	α5 (JM1)
Q133A	0.25 ± 0.062	α5 (JM1)
F134A	0.48 ± 0.023	α5 (JM1)
T135A	0.32 ± 0.031	α5 (JM1)
A136M	0.023 ± 0.0029	α5 (JM1)
K137A	0.43 ± 0.057	α5 (JM1)
M138A	0.84 ± 0.057	α5 (JM1)
F139A	0.13 ± 0.0032	α5 (JM1)
Y140A	0.083 ± 0.0096	α5 (JM1)
Y140F	0.042 ± 0.0018	α5 (JM1)
K141A	0.50 ± 0.020	α5 (JM1)
V142A	0.40 ± 0.020	α5 (JM1)
I143A	0.97 ± 0.054	α5 (JM1)
G144A	1.4 ± 0.068	α5 (JM1)
R145A	6.7 ± 0.20	α5-β6 loop
M146A	0.82 ± 0.040	α5-β6 loop
D157N	0.018 ± 0.0029	α5-β6 loop
R200Q	0.010 ± 0.00076	β7-α7 loop
T205P	0.20 ± 0.091	β7-α7 loop
Y209A	0.18 ± 0.027	α7 (JM2)
W210A	0.16 ± 0.013	α7 (JM2)
K211A	0.54 ± 0.024	α7 (JM2)
L212A	0.097 ± 0.0044	α7 (JM2)
L212F	0.027 ± 0.0021	α7 (JM2)
W213A	0.087 ± 0.010	α7 (JM2)
N214A	0.27 ± 0.027	α7 (JM2)
F215A	0.19 ± 0.044	α7 (JM2)
A216M	0.039 ± 0.0014	α7 (JM2)
L217A	0.17 ± 0.064	α7 (JM2)
D218A	0.40 ± 0.026	α7 (JM2)
G219A	0.15 ± 0.013	α7 (JM2)
I220A	0.13 ± 0.0089	α7 (JM2)
F221A	37 ± 0.26	α7 (JM2)
S222A	0.99 ± 0.032	α7 (JM2)
F223A	1.4 ± 0.014	α8 (TM1)
S224A	1.4 ± 0.026	α8 (TM1)
L225A	3.3 ± 0.16	α8 (TM1)
L226A	2.3 ± 0.036	α8 (TM1)
R290Q	0.021 ± 0.0016	α9 (TM2)

Supplementary Table 1 | Comprehensive list of GtrBSyn mutants made and tested for this study, inclusive of their activity level and location on the structure.

	Forward	Reverse
**dpm1 ex1-2**	5'-GTTGATCGTCTGGTTGCTGG-3'	5'- GCGCCGTAAATCTTCTGCAGC-3'
**eifalpha**	5'-CAGCTGATCGTTGGAGTCAA-3'	5'-TCTTCCATCCCTTGAACCAG-3'
**rpl8**	5'-GGCTAAGGTGATGTTTTCGTG-3'	5'-GCACATTGCCAATGTTCAGC-3'

Supplementary Table 2 | Zebrafish qPCR primers used for this study.

Supplementary References

1. Kyte, J. & Doolittle, R. F. A simple method for displaying the hydropathic character of a protein. *J. Mol. Biol.* **157**, 105–132 (1982).
2. Pedersen, L. C., Tsuchida, K., Kitagawa. H., Sugahara, K., Darden, T. A., Negishi, M. Heparan/chondroitin sulfate biosynthesis. Structure and mechanism of human glucuronyltransferase I. *J Biol Chem.* **257**, 34580-34585 (2000).
3. Nguyen, H. P., Seto, N. O., Cai, Y., Leinala, E. K., Borisova, S. N., Palcic, M. M., Evans, S. V. The influence of an intramolecular hydrogen bond in differential recognition of inhibitory acceptor analogs by human ABO(H) blood group A and B glycosyltransferases. *J Biol Chem.* **278**, 49191-49195. (2003).
4. Nielsen, M. M., Suits, M. D., Yang, M., Barry, C. S, Martinez-Fleites, C., Tailford, L. E., Flint, J. E., Dumon, C., Davis, B. G., Gilbert, H. J., Davies, G. J. Substrate and metal ion promiscuity in mannosylglycerate synthase. *J Biol Chem.* **286**, 15155-15164 (2011).
5. Lomize, M. A., Pogozheva, I. D. , Joo, H., Mosberg, H. I., Lomize, A. L., OPM database and PPM web server: resources for positioning of proteins in membranes. *Nucleic Acids Res*. **40** (Database issue), D370-6 (2012).
